# OX40 (TNFRSF4) Tracks Immune Infiltration in Small Cell Lung Cancer and Shows a Cohort-Specific, Non-Replicated Association with the POU2F3/SCLC-P Subtype: A Multi-Source In Silico Analysis

**DOI:** 10.3390/ijms27177823

**Published:** 2026-08-31

**Authors:** Mehmet Turan Özer, Faruk Recep Özalp

**Affiliations:** 1 Department of Internal Medicine, Osmaniye Training and Research Hospital, 80010 Osmaniye, Türkiye; 2 Department of Medical Oncology, Faculty of Medicine, Dokuz Eylül University, 35340 İzmir, Türkiye; ozalpfarukrecep@gmail.com

**Keywords:** OX40, TNFRSF4, small cell lung cancer, SCLC-P, POU2F3, tumour immune microenvironment, immune infiltration, molecular subtype, in silico analysis, biomarker hypothesis

## Abstract

OX40 (TNFRSF4) is a costimulatory T-cell receptor and OX40 agonists are in clinical development, yet its role in small cell lung cancer (SCLC) is still to be characterised. We analysed the discovery cohort (*n* = 77, 48 events), GSE60052 (*n* = 79 tumours; 48 with survival), GDSC1+GDSC2 (61 SCLC cell lines, 542 drugs) and human SCLC single-cell atlas (77,143 cells from primary and metastatic sites, 20 donors), using Cox models, FDR-controlled correlation, nested-model comparison and deconvolution. High TNFRSF4 showed a non-significant protective trend (pooled HR = 0.86 per SD, 0.68–1.10, *p* = 0.23); a nominal cutpoint did not survive correction for cutpoint search (*p* = 0.25). No drug reached FDR < 0.05 among 658 tests, including platinum agents and etoposide. TNFRSF4 tracked immune infiltration (13-gene score ρ = 0.76, *p* = 3 × 10^−16^) and was detected in 17.6% of T cells versus 1.03% of malignant cells in all 20 donors. OX40 was enriched in the POU2F3/SCLC-P subtype (*p* = 0.033), persisting after immune adjustment (β = 1.32, *p* = 0.011) but not replicating independently (*p* = 0.10). Adding TNFRSF4 to clinical-plus-immune models provided no meaningful discrimination gain (ΔC-index ≤ 0.005). Power was limited to HR ≥ 1.50 harmful or HR ≤ 0.67 protective, so the observed trend is undetectable at this size. Bulk OX40 therefore primarily reflects immune infiltration, and its SCLC-P association is a hypothesis for prospective testing.

## 1. Introduction

Small cell lung cancer (SCLC) accounts for roughly 13–15% of lung cancers and remains the most aggressive thoracic malignancy, with a five-year survival below 7% that reflects early metastatic dissemination and rapidly acquired chemoresistance [1,2]. Platinum-etoposide chemotherapy has been the backbone of first-line treatment for four decades, and the addition of PD-L1 blockade to chemotherapy in extensive-stage disease extends median overall survival by only two to three months [3]. The magnitude of benefit from PD-1 axis inhibition in SCLC is smaller than in most other solid tumours and a durable effect is confined to a minority of patients, plausibly because SCLC combines T-cell exclusion, downregulation of MHC class I antigen processing and presentation, and upregulation of inhibitory myeloid checkpoints [4]. No predictive biomarker is established in this setting, and patient selection remains the principal unmet need in SCLC immunotherapy [5]. This has motivated a search for costimulatory targets that could be combined with, or substituted for, PD-1/PD-L1 blockade, and for markers capable of identifying the patients in whom such targets are actually engaged.

OX40 (TNFRSF4, CD134) is a costimulatory receptor of the tumour-necrosis-factor-receptor superfamily, expressed transiently on recently activated CD4+ and CD8+ T cells and constitutively on regulatory T cells. Engagement by its ligand OX40L activates NF-κB and PI3K/Akt signalling cascades, promoting clonal expansion and survival of effector T cells while inhibiting regulatory T-cell function, a dual mechanism that makes agonistic OX40 antibodies attractive as immunotherapy [6,7,8].

The clinical experience with OX40 agonism illustrates why a marker of target relevance is needed. Early-phase clinical trials of OX40 agonists have demonstrated limited efficacy despite evidence of target engagement, highlighting the need for reliable biomarkers to guide patient selection [9,10,11,12]. Occupying the receptor is therefore not sufficient, and the outstanding question is in which tumours, and in which cellular compartment, OX40 expression carries information that could inform patient selection.

Bulk TNFRSF4 expression has been proposed as such an informative marker in several malignancies. Previous studies across several malignancies have reported heterogeneous associations between TNFRSF4 expression, immune infiltration and prognosis, suggesting that its biomarker value is context-dependent [13,14,15,16], and reviews of the field reach the same conclusion [8]. A recurring methodological feature of these transcriptomic studies is that the prognostic association and the correlation with immune-cell abundance are reported alongside one another rather than tested against one another, leaving open whether OX40 carries prognostic information of its own or is a readout of how many lymphocytes are present. In SCLC, OX40-positive tumour-infiltrating lymphocytes and OX40/OX40L expression have been associated with favourable outcomes [17,18], within a wider literature reporting prognostic value for tumour OX40 expression across tumour types [19,20]. Whether bulk TNFRSF4 expression in SCLC provides prognostic information beyond immune infiltration, associates independently with molecular subtype, or reflects tumour-intrinsic drug sensitivity has not been systematically evaluated.

The transcriptional taxonomy of SCLC provides a natural framework for such a question. Four subtypes defined by the dominant lineage transcription factor, namely SCLC-A (ASCL1), SCLC-N (NEUROD1), SCLC-P (POU2F3) and SCLC-Y (YAP1), differ in therapeutic vulnerability, and a revised scheme replaced the poorly reproducible YAP1 group with an immune-inflamed SCLC-I state that is enriched among patients benefiting from checkpoint blockade [21,22]. The POU2F3-driven SCLC-P subtype lacks neuroendocrine features, depends on distinct lineage factors, and has no established targeted option, making it a subgroup of particular interest for immunotherapy hypotheses [21]. Lineage identity in SCLC is itself plastic rather than fixed, which limits how far any single subtype assignment can be pushed [2].

Any expression-based biomarker in bulk SCLC tissue faces a specific confound; immune genes are expressed predominantly by infiltrating leukocytes, so a bulk signal may primarily reflect the abundance of immune cells rather than a property of the tumour. Distinguishing these possibilities requires that a candidate be tested against a quantitative infiltration score, that its prognostic contribution be assessed beyond what infiltration and standard clinical variables already provide, and that its cellular source be resolved at the single-cell level. We therefore analysed OX40 across four complementary public sources, asking whether TNFRSF4 expression carries independent prognostic information, predicts sensitivity to agents used in SCLC, marks a molecular subtype, and originates from tumour or immune cells. Because several of the central results are null, we also quantified the effect sizes that could have been detected in each cohort, so that the boundary between an absent and an undetected association is explicit.

## 2. Results

### 2.1. No Independent Prognostic Association Was Detectable in Either Cohort

**Figure 1:**
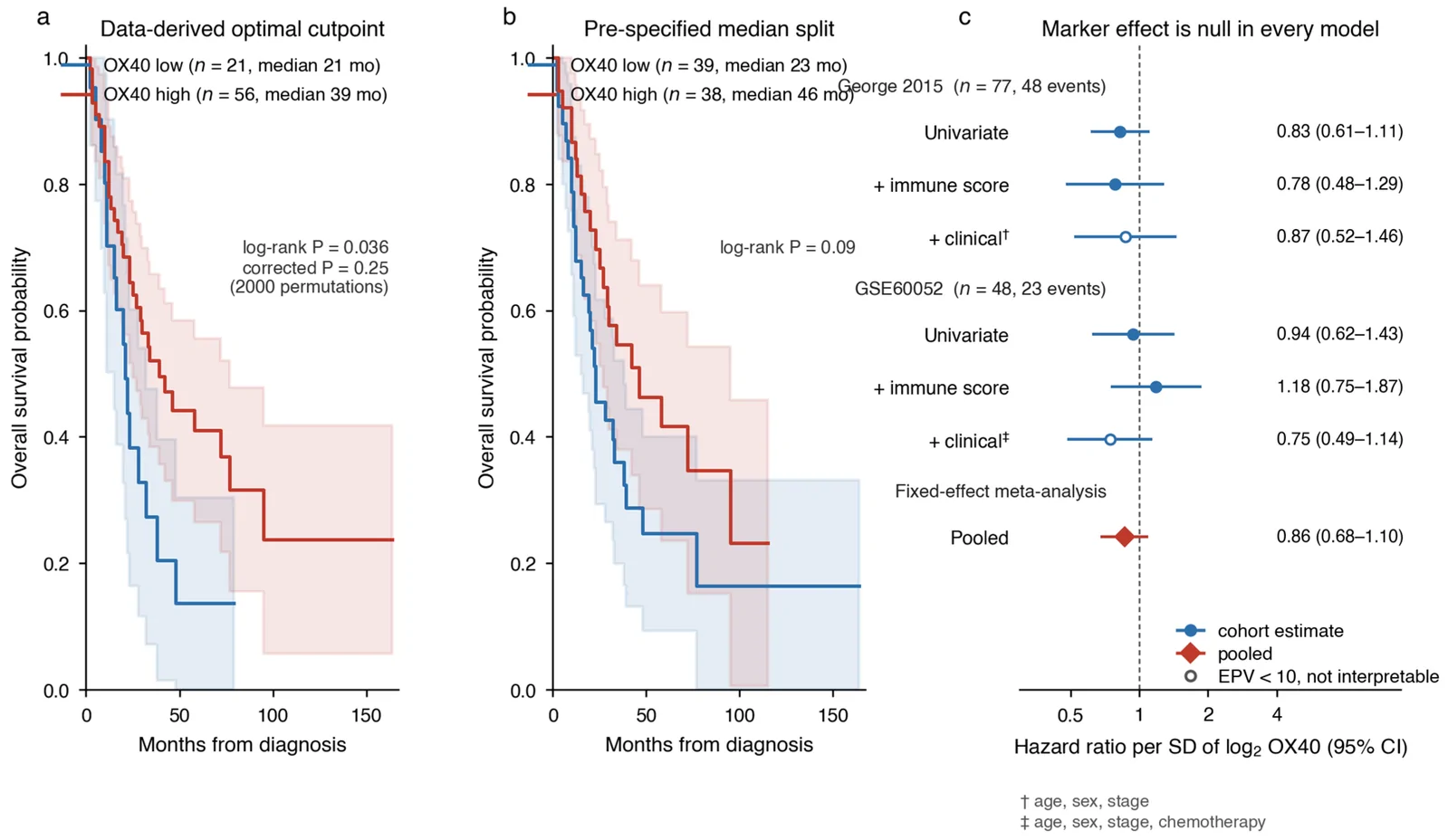
OX40/TNFRSF4 and overall survival. (**a**) Kaplan–Meier curves for the data-derived optimal cutpoint in George 2015 [23] (*n* = 77, 48 events); the uncorrected log-rank P is shown together with the P value after 2000 permutations of the entire cutpoint search. (**b**) Kaplan–Meier curves for the pre-specified median split in the same cohort. (**c**) Forest plot of harmonised Cox hazard ratios per standard deviation of log2 TNFRSF4 for George 2015 [23], GSE60052 and a fixed-effect meta-analysis, showing univariable, immune-adjusted, clinically adjusted and fully adjusted (clinical plus immune) models in both cohorts. Open markers with dashed intervals denote models with fewer than ten events per variable, which are overfitted and not interpretable as adjusted effects. † age, sex, stage; ‡ age, sex, stage, chemotherapy exposure. Shaded bands in (**a**,**b**) are 95% confidence bands. In (**c**), red denotes the pooled fixed-effect estimate and blue the individual cohort estimates; open circles with dashed intervals denote models with fewer than ten events per variable.

In the George et al. [23] (*n* = 77, 48 events), a data-derived optimal cutpoint separated 56 OX40-high from 21 OX40-low patients with nominally significant survival difference (median 39 versus 21 months, log-rank *p* = 0.036; Figure 1a). This cutpoint was selected by scanning all candidate thresholds, and after a permutation procedure that repeats the entire search on 2000 label permutations, the association was no longer significant (*p* = 0.25). The pre-specified median split, which involves no selection, gave a concordant but non-significant separation (46 versus 23 months, *p* = 0.086; Figure 1b). We therefore treat the cutpoint result as an artefact of threshold optimisation and report continuous per-SD estimates throughout.

Across harmonised Cox models, both univariable cohort estimates and the pooled estimate were below 1, whereas adjusted estimates were directionally inconsistent, and no model reached statistical significance (Figure 1c). In George 2015 [23], TNFRSF4 gave HR = 0.83 per SD (95% CI 0.61–1.11, *p* = 0.21) univariably, HR = 0.78 (0.48–1.29, *p* = 0.33) after adjustment for the immune infiltration score, and HR = 0.91 (0.66–1.24, *p* = 0.53) after adjustment for age, sex and stage, and HR = 0.87 (0.52–1.46, *p* = 0.60) after adjustment for both clinical covariates and the immune score. In GSE60052 (*n* = 48 with survival, 23 events), the univariable estimate was HR = 0.94 (0.62–1.43, *p* = 0.78), the immune-adjusted estimate HR = 1.18 (0.75–1.87, *p* = 0.47), the clinically adjusted estimate HR = 0.75 (0.49–1.14, *p* = 0.18) and the estimate adjusted for both HR = 1.08 (0.63–1.85, *p* = 0.79). Clinical adjustment in this cohort additionally included chemotherapy exposure. The two fully adjusted models fall below ten events per variable and are reported as flagged, non-interpretable estimates. A fixed-effect meta-analysis of the two univariable estimates gave a pooled HR = 0.86 (0.68–1.10, *p* = 0.23).

Two features of these models require explicit qualification. First, the fully adjusted models are overfitted relative to the available events; the clinically adjusted George 2015 [23] model had 12 events per variable, whereas its fully adjusted clinical-plus-immune model had 9.6; in GSE60052 the clinically adjusted and fully adjusted models had 4.6 and 3.8 events per variable, respectively. Models falling below the conventional threshold of ten are flagged in Figure 1c and should not be interpreted as reliable adjusted effects. Second, the variables that did reach significance were the expected clinical and immunological ones rather than OX40: male sex in George 2015 [23] (HR = 2.85, 1.32–6.17, *p* = 0.008) and stage III-IV in GSE60052 (HR = 9.60, 2.48–37.2, *p* = 0.001). Notably, the immune infiltration score itself was prognostic in GSE60052 (HR = 0.52 per SD, 0.32–0.84, *p* = 0.007), while TNFRSF4 was not in the same model, an early indication that the infiltration signal rather than OX40 carries the survival information. Kaplan–Meier curves and the full set of hazard-ratio estimates for the validation cohort, together with the pooled estimate, are shown in Figure S1; TNFRSF4 model estimates are provided in Supplementary Table S1, and full covariate output is provided in File S3.

### 2.2. OX40 Expression Does Not Predict Sensitivity to Any Drug in the SCLC Cell-Line Panel

**Figure 2:**
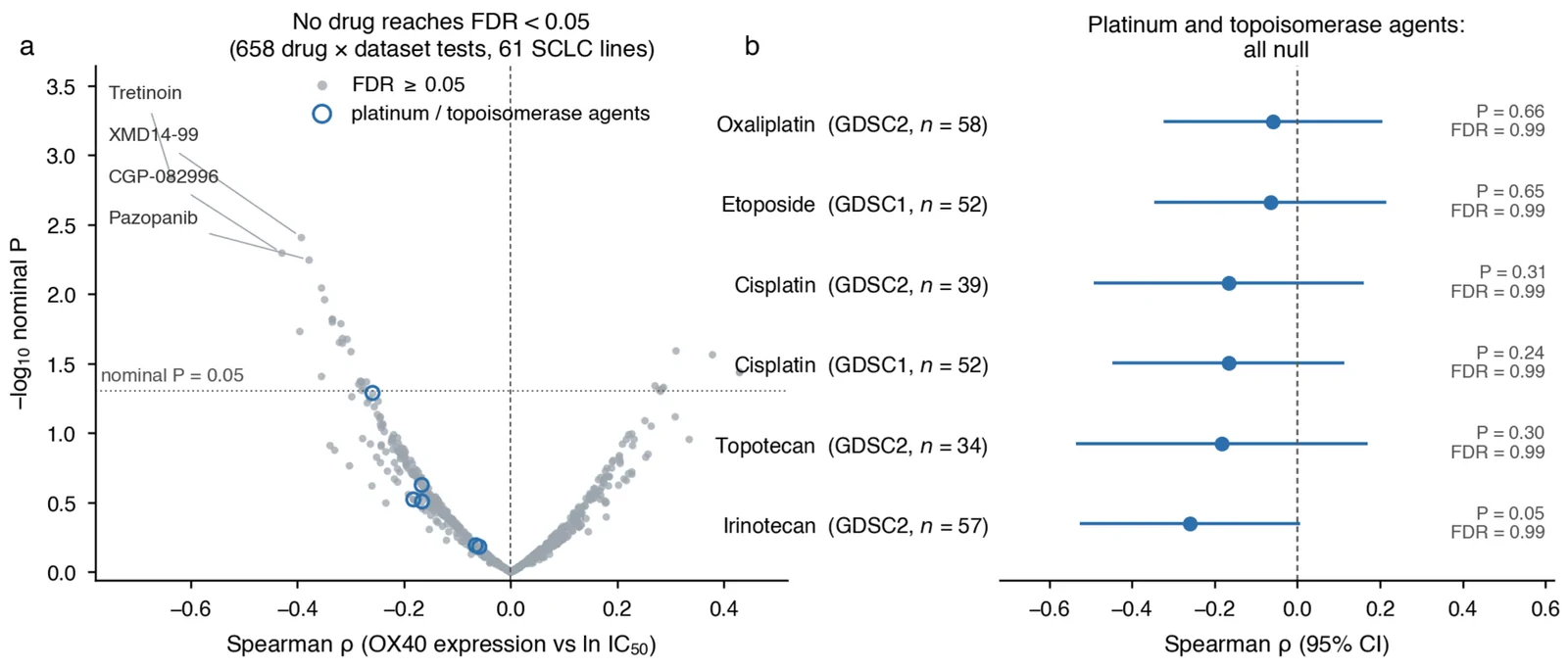
TNFRSF4 expression and drug sensitivity in SCLC cell lines (GDSC1+GDSC2; 61 lines, 542 compounds, 658 drug×dataset tests, one observation per cell line). (**a**) Volcano plot of Spearman correlation between log2 TNFRSF4 and ln(IC50) against significance; no compound reached FDR < 0.05 (minimum FDR = 0.93) and the dashed line marks the nominal *p* = 0.05 threshold. (**b**) Correlation coefficients with 95% confidence intervals for the pre-specified agents used clinically in SCLC. Negative coefficients indicate greater sensitivity with higher TNFRSF4. All intervals cross zero.

Across 61 SCLC cell lines and 542 compounds, 658 drug×dataset correlations between TNFRSF4 expression and ln(IC50) were computed after collapsing replicate curves to one observation per cell line (full results in Supplementary Table S2). No association survived multiplicity control: the smallest Benjamini–Hochberg FDR across the entire panel was 0.93 (Figure 2a). The lowest raw *p*-value belonged to tretinoin (ρ = −0.64, *p* = 0.0014, *n* = 22 lines), which is neither used in SCLC nor plausibly linked to OX40 biology, and is best read as the expected extreme of 658 tests.

The pre-specified agents used clinically in SCLC were uniformly negative (Figure 2b). All five drug×dataset combinations pointed in the direction of greater sensitivity with higher TNFRSF4, but none approached significance: irinotecan ρ = −0.26 (*p* = 0.052, *n* = 57), cisplatin ρ = −0.17 in both GDSC1 (*p* = 0.24, *n* = 52) and GDSC2 (*p* = 0.31, *n* = 39), topotecan ρ = −0.18 (*p* = 0.30, *n* = 34), and etoposide ρ = −0.07 (*p* = 0.65, *n* = 52).

This null result has a straightforward biological explanation rather than being merely uninformative. GDSC cell lines are pure tumour cultures without an immune compartment, so any TNFRSF4 signal they carry must be tumour-intrinsic. The absence of such a signal, together with the single-cell attribution in Section 2.4, indicates that bulk TNFRSF4 expression predominantly reflects the tumour microenvironment; the cell-line analysis does not establish whether immune-mediated mechanisms influence treatment response in patients. A seven-gene T-cell costimulatory-axis signature (TNFRSF4, TNFRSF9, TNFRSF18, TNFSF4, ICOS, CD27, TNFRSF14; the list spans TNF-receptor-superfamily members, the OX40 ligand TNFSF4 and the CD28-family receptor ICOS) reproduced the same null pattern (Figure S2, Supplementary Table S3), indicating that the result is not an artefact of relying on a single gene.

### 2.3. OX40 Is a Strong Surrogate for Tumour Immune Infiltration

**Figure 3:**
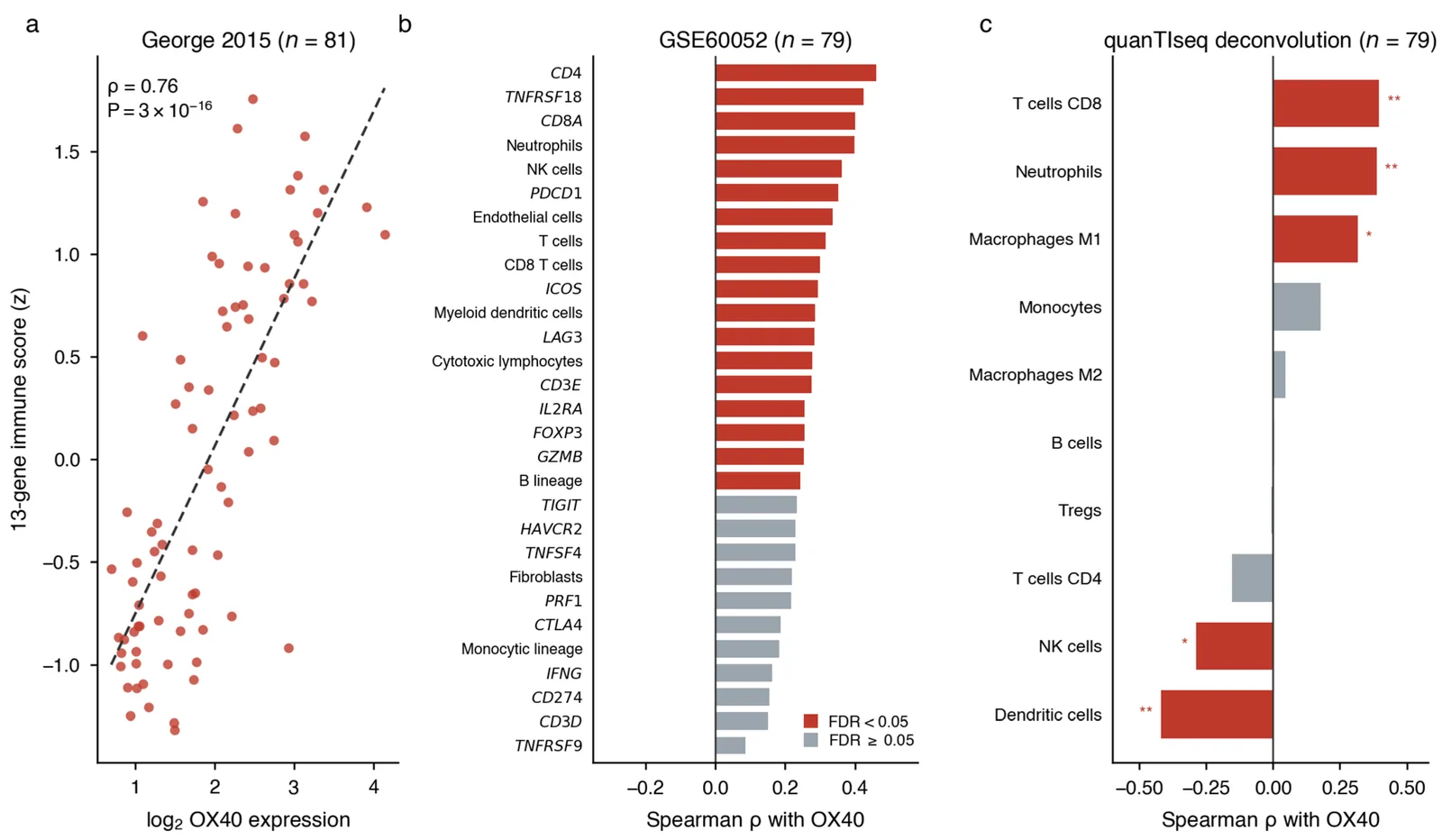
TNFRSF4 as a surrogate for immune infiltration. (**a**) TNFRSF4 versus the 13-gene immune score in George 2015 [23] (*n* = 81) with fitted trend; Spearman ρ and *p* are shown. (**b**) Spearman correlations between TNFRSF4 and immune marker genes and MCP-counter population scores in GSE60052 (*n* = 79); features significant at FDR < 0.05 are highlighted and 18 of 29 features are positive. (**c**) Correlations with quanTIseq-derived cell-type fractions in the same cohort. Because these fractions are compositional and dominated by an uncharacterised residual compartment (mean 76% of each sample), negative coefficients are interpreted as compositional constraints rather than depletion, and all quanTIseq results are exploratory. Asterisks in (**c**) denote the Benjamini-Hochberg false discovery rate: * *p* < 0.05, ** *p* < 0.01. In (**b**), red bars denote features significant at FDR < 0.05 and grey bars features that are not; gene symbols are italicised and MCP-counter population scores are not.

TNFRSF4 expression tracked immune infiltration closely in both cohorts. In George 2015 [23], a 13-gene immune score correlated with TNFRSF4 at ρ = 0.76 (*p* = 3 × 10^−16^, *n* = 81), an association strong enough that their strong correlation reduces the precision of their jointly estimated effects (Figure 3a). In GSE60052, 18 of 29 immune features were positively correlated at FDR < 0.05, including CD4 (ρ = 0.46, FDR = 5 × 10^−4^), TNFRSF18 (ρ = 0.43, FDR = 0.001), CD8A (ρ = 0.40, FDR = 0.002), PDCD1/PD-1 (ρ = 0.35, FDR = 0.007), the MCP-counter T-cell score (ρ = 0.32, FDR = 0.017) and FOXP3 (ρ = 0.26, FDR = 0.041), while CTLA4 and CD274/PD-L1 did not reach significance (Figure 3b). Co-expression with TNFRSF18/GITR, the closest costimulatory relative of OX40, is consistent with both genes marking the same activated T-cell population.

An independent deconvolution approach gave a concordant picture (Figure 3c). Applying quanTIseq to the GSE60052 profiles, TNFRSF4 correlated positively with CD8+ T-cell (ρ = 0.40, FDR = 0.001), neutrophil (ρ = 0.39, FDR = 0.001) and M1 macrophage (ρ = 0.32, FDR = 0.011) estimates, and negatively with dendritic-cell (ρ = −0.42, FDR = 0.001) and NK-cell (ρ = −0.29, FDR = 0.020) estimates. These fractions are compositional and are dominated by an uncharacterised residual compartment that accounts for a mean of 76% of each sample, so the negative coefficients most plausibly reflect the constraint that fractions sum to one rather than genuine depletion of those populations. We therefore treat the deconvolution as corroborating the marker-gene analysis for CD8+ T cells and as exploratory for all other cell types. Complete correlation tables are provided in Supplementary Tables S4 and S5.

### 2.4. Single-Cell Analysis Localises OX40 to T Cells Rather than Malignant Cells

**Figure 4:**
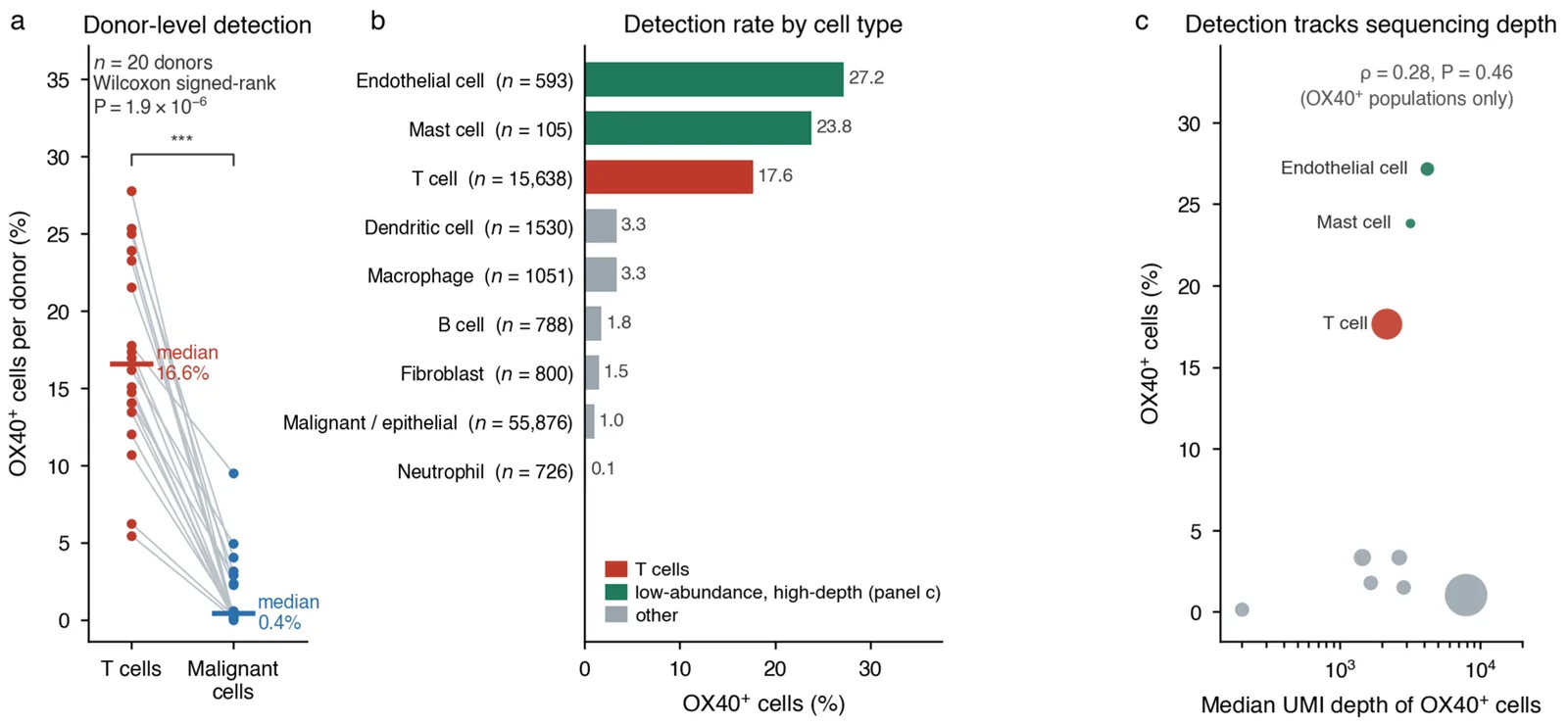
Single-cell attribution of TNFRSF4 expression (human SCLC atlas; 77,143 cells from primary and metastatic sites, 20 donors). (**a**) Paired donor-level percentage of TNFRSF4-positive T cells versus malignant cells; each grey line joins the two values for one donor and all 20 donors separate in the same direction (paired Wilcoxon signed-rank *p* = 1.9 × 10^−6^, raw *p* value, no multiplicity adjustment applied). Red denotes T cells and blue malignant/epithelial cells; horizontal bars mark group medians. *** *p* < 0.001. (**b**) Pooled positivity by annotated cell type for the nine populations with at least 100 cells, with the percentage and the number of cells in each population given beside each bar. Red denotes T cells, green the two low-abundance high-depth populations highlighted in (**c**) and grey all other populations. (**c**) Median sequencing depth of each population versus its positivity, with marker area proportional to the number of cells in the population; malignant epithelial cells have the highest depth of any population, so their low positivity is not a depth artefact, and across populations depth and positivity are not significantly correlated (Spearman ρ = 0.28, *p* = 0.46). Endothelial and mast-cell estimates rest on 593 and 105 cells, respectively, and are exploratory. OX40+ denotes TNFRSF4-positive cells.

To determine whether the bulk-tissue signal originates from tumour or immune cells, TNFRSF4 detection was quantified in 77,143 cells from 20 donors in a human SCLC atlas spanning primary and metastatic sites. At the donor level, the separation was unambiguous and fully consistent; median 16.6% of T cells were TNFRSF4-positive versus 0.43% of malignant cells, a mean 10.4-fold difference, and every one of the 20 donors had higher positivity in T cells than in malignant cells (Wilcoxon signed-rank *p* = 1.9 × 10^−6^; Figure 4a). Pooled across donors, 17.6% of 15,638 T cells were positive compared with 1.03% of 55,876 malignant epithelial cells (Figure 4b).

Two observations qualify the cell-type ranking. Endothelial cells (27.2% of 593 cells) and mast cells (23.8% of 105 cells) had the highest positivity rates of any annotated population, exceeding T cells. Functional endothelial OX40 expression has been reported in other tumour types, where endothelial OX40 activation promotes S1P/YAP-mediated angiogenesis and tumour-cell escape from T-cell surveillance, with an endothelial positivity of 26.75% closely comparable to the 27.2% observed here [24]. A genuine endothelial contribution therefore cannot be excluded, and the following observations bound rather than dismiss it. Endothelial cells showed higher raw per-cell counts than T cells (mean 1.04 versus 0.48 TNFRSF4 counts per cell; 3.84 versus 2.74 among positive cells; Supplementary Table S6), but these cells were also sequenced more deeply (median 4197 versus 2162 unique molecular identifiers), and after normalisation for depth the ordering reverses: median expression among positive cells was 341 CPM in endothelial cells versus 597 CPM in T cells (Mann–Whitney *p* = 2.4 × 10^−13^ in favour of T cells). The excess is therefore confined to detection frequency, which remains elevated after depth adjustment (odds ratio 1.28, 1.05–1.56, *p* = 0.015), while the donor-paired comparison of detection was not significant (Wilcoxon *p* = 0.094, 13 evaluable donors; Figure S4). Restricting the pairing to the 11 donors contributing at least 20 cells to both compartments gives the same answer (endothelial median 20.0% versus T-cell 17.0% positivity, paired Wilcoxon *p* = 0.32; mean counts per cell 0.77 versus 0.44, *p* = 0.83; Supplementary Table S13), so the endothelial excess is not statistically supported at donor level, is limited by the small number of endothelial cells, and is reported as an exploratory finding requiring independent validation. Taken together, endothelial TNFRSF4 detection exceeded that in T cells and remained elevated after adjustment for sequencing depth, but the limited endothelial sample, the donor-level variability and the absence of orthogonal protein validation prevent determination of whether this signal represents functional endothelial OX40 expression in SCLC or low-level detection arising from ambient RNA, doublets or physiological background expression. More importantly, detection rates in droplet-based single-cell data depend on sequencing depth, and the populations compared here differ substantially in median depth (Figure 4c). Malignant epithelial cells had the highest median depth of any population (7921 counts versus 2162 in T cells), so their low positivity cannot be attributed to shallow sequencing, and if anything, the comparison is conservative. Across populations with at least 100 cells and non-zero detection, depth and positivity were not significantly correlated (ρ = 0.28, *p* = 0.46), and the malignant-T-cell contrast was essentially unchanged in a depth-adjusted logistic model (Supplementary Table S7). The T-cell versus malignant-cell contrast is therefore robust, while the absolute positivity rates should be considered lower bounds given the sparsity of single-cell counts. Detection rates for all 11 annotated populations are shown in Figure S3 and tabulated in Supplementary Table S6.

### 2.5. OX40 Is Enriched in the POU2F3/SCLC-P Subtype but the Association Does Not Replicate

**Figure 5:**
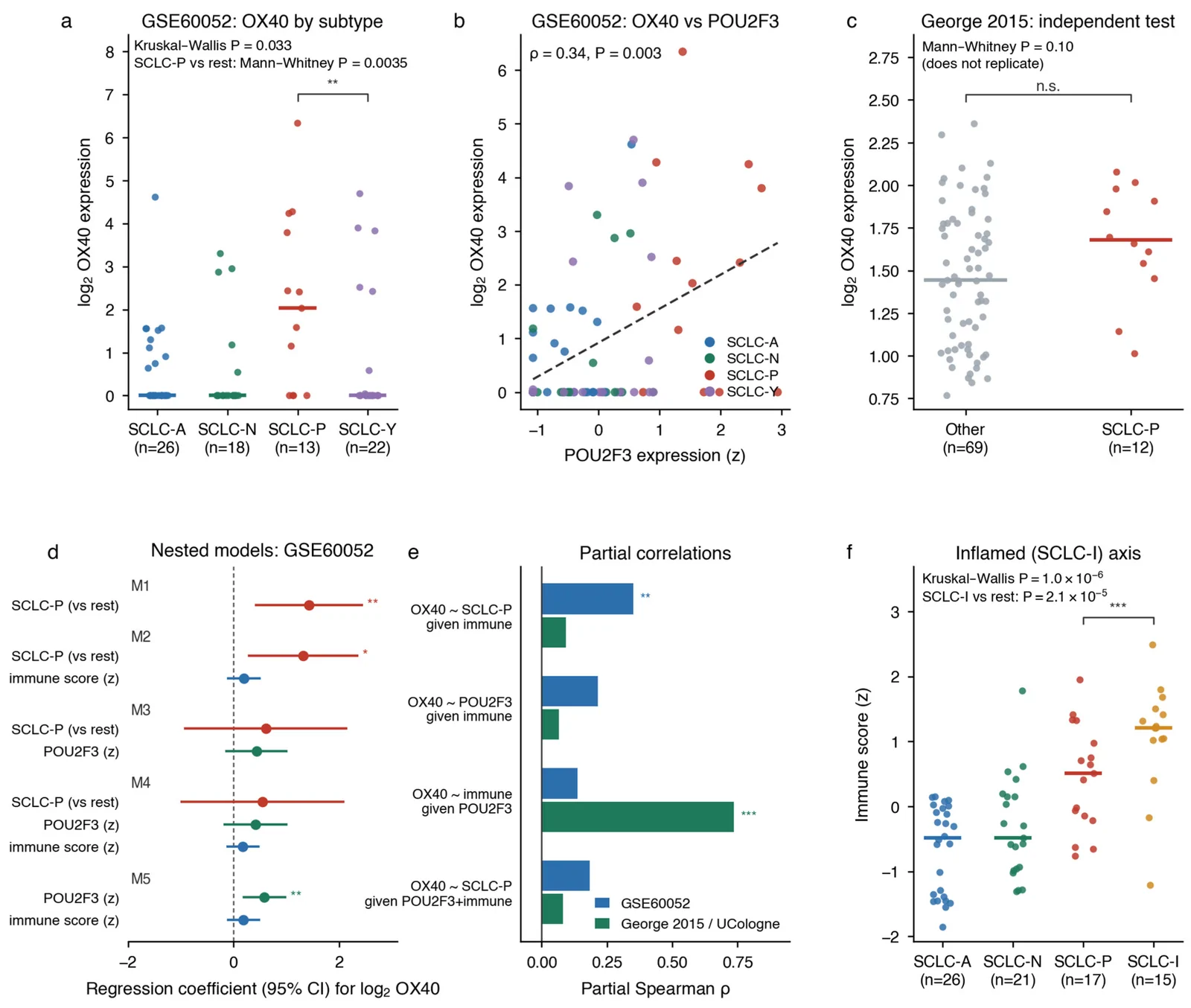
TNFRSF4 and molecular subtype. (**a**) TNFRSF4 across the four-way ASCL1/NEUROD1/POU2F3/YAP1 subtypes in GSE60052 (*n* = 79); horizontal lines mark medians. (**b**) TNFRSF4 versus continuous POU2F3 expression. (**c**) Independent subtype replication in the George 2015 [23]/UCologne cohort (*n* = 81) under the four-way lineage-defined classification; 13 SCLC-P tumours versus 68 others (Mann–Whitney *p* = 0.038). (**d**) Regression coefficients for the SCLC-P indicator and continuous POU2F3 entered separately and together; the two are collinear (r = 0.77, variance inflation factor 2.50) and neither survives joint entry. (**e**) Partial Spearman correlations in the GSE60052 and George 2015 [23]/UCologne cohorts after adjustment for immune infiltration and lineage-related variables; the SCLC-P association is present only in GSE60052, whereas the infiltration association dominates in George 2015 [23]. (**f**) Immune score and TNFRSF4 across the revised inflamed-axis subtypes; SCLC-I is strongly immune-enriched but shows no TNFRSF4 elevation. Colours denote molecular subtype in (**a**,**b**,**f**): blue denotes SCLC-A, green denotes SCLC-N, red denotes SCLC-P, purple denotes SCLC-Y and ochre denotes SCLC-I. In (**c**), grey denotes the other subtypes and red denotes SCLC-P. In (**d**), red denotes the SCLC-P indicator, green denotes continuous POU2F3 and blue denotes the immune score; the coloured horizontal lines show the corresponding 95% confidence intervals. In (**e**), blue denotes GSE60052 and green denotes George 2015 [23]/UCologne. Coloured asterisks refer to the estimate shown in the corresponding colour: * *p* < 0.05; ** *p* < 0.01; *** *p* < 0.001. Dashed lines mark the null value (zero) in (**d**) and the fitted trend in (**b**); horizontal lines in (**a**,**c**,**f**) mark group medians.

Assigning GSE60052 tumours to the four-way ASCL1/NEUROD1/POU2F3/YAP1 scheme yielded SCLC-A (*n* = 26), SCLC-N (*n* = 18), SCLC-P (*n* = 13) and SCLC-Y (*n* = 22). TNFRSF4 was markedly higher in SCLC-P (median 2.03 versus 0.00 log2 units in the other subtypes; Kruskal–Wallis *p* = 0.033, SCLC-P versus rest *p* = 0.0035; Figure 5a; the corresponding value under the inflamed-axis assignment, which places 17 rather than 13 tumours in SCLC-P, is *p* = 0.0023 and is reported in Supplementary Table S8), and continuous POU2F3 expression correlated with TNFRSF4 across all tumours (ρ = 0.34, *p* = 0.003; Figure 5b).

This enrichment was not explained by immune infiltration. In a linear model containing both the SCLC-P indicator and the immune score, SCLC-P retained a substantial coefficient (β = 1.32, 95% CI 0.30–2.34, *p* = 0.011) while the immune term did not contribute (*p* = 0.20), and the estimate was stable under heteroscedasticity-robust standard errors, 10,000 bootstrap resamples (95% percentile interval 0.36–2.33) and 10,000 label permutations (*p* = 0.001). The partial Spearman correlation between TNFRSF4 and SCLC-P status controlling for infiltration was ρ = 0.35 (*p* = 0.002; Figure 5e). The subtype signal is thus separable from the infiltration signal that dominates Section 2.1 and Section 2.3.

Three findings nonetheless constrain how far this can be taken. First, the SCLC-P indicator is not separable from continuous POU2F3 expression; the two are strongly collinear (r = 0.77, variance inflation factor 2.50), and when both are entered together neither remains significant, whereas continuous POU2F3 alone is a significant predictor (β = 0.59, 0.19–0.98, *p* = 0.003; Figure 5d). The signal is best described as tracking POU2F3-driven biology on a continuum rather than as a property of a discrete subtype. Second, the enrichment did not replicate in the independent George 2015 [23]/UCologne cohort, where 13 SCLC-P tumours had no significant elevation (median 2.20 versus 1.72 log2 FPKM, *p* = 0.10; label permutation *p* = 0.14), and the partial correlation with SCLC-P status fell to ρ = 0.09 (*p* = 0.41; Supplementary Table S8) while the partial correlation with infiltration remained ρ = 0.74 (*p* = 7.4 × 10^−15^), the reverse of the GSE60052 pattern (Figure 5e). The replication test is sensitive to how subtypes are assigned in this cohort. Under the four-way ASCL1/NEUROD1/POU2F3/YAP1 scheme, the George 2015 [23] tumours fall into SCLC-A (*n* = 25), SCLC-N (*n* = 22), SCLC-P (*n* = 13) and SCLC-Y (*n* = 21). TNFRSF4 then differs across groups (Kruskal–Wallis *p* = 2.1 × 10^−4^) with nominal elevation in SCLC-P (*p* = 0.038; Figure 5c). Under the inflamed-axis scheme, in which the YAP1 group is replaced by an immune-defined SCLC-I state, the same comparison gives *p* = 0.10. Because the inflamed-axis assignment is itself built from immune expression, it is not an independent test for a gene that tracks infiltration, and the four-way scheme is the appropriate comparator. The nominal four-way result does not survive the necessary adjustment, with the pan-immune score in the model the SCLC-P coefficient falls to β = 0.24 (95% CI −0.09 to 0.56, *p* = 0.15), continuous POU2F3 adjusted for the other lineage factors and infiltration is not significant (β = 0.07, 95% CI −0.09 to 0.23, *p* = 0.38), and a POU2F3-target tuft-cell signature is likewise uninformative after immune adjustment (β = 0.05, −0.07 to 0.17, *p* = 0.42; partial ρ = 0.13, *p* = 0.23) whereas in GSE60052 the same signature remains strongly associated (β = 1.07, 0.59 to 1.56, *p* = 3.7 × 10^−5^; partial ρ = 0.38, *p* = 4.7 × 10^−4^). The enrichment therefore fails to replicate on either assignment once infiltration is accounted for (Supplementary Table S12). Third, under the revised inflamed-axis scheme (SCLC-A *n* = 26, SCLC-N *n* = 21, SCLC-P *n* = 17, SCLC-I *n* = 15), the SCLC-I group showed the expected strong elevation of immune score (Kruskal–Wallis *p* = 1.0 × 10^−6^; SCLC-I versus rest *p* = 2.1 × 10^−5^) but no elevation of TNFRSF4 (*p* = 0.35; Figure 5f). OX40 therefore does not mark the inflamed state, which is an informative dissociation. It separates the OX40/SCLC-P signal from the broad immune axis and suggests that the two are distinct phenomena. Taken together, the SCLC-P association is internally robust in the discovery cohort but unreplicated, and is presented as a hypothesis rather than an established subtype marker.

### 2.6. OX40 Adds No Prognostic Information Beyond Clinical and Immune Variables

**Figure 6:**
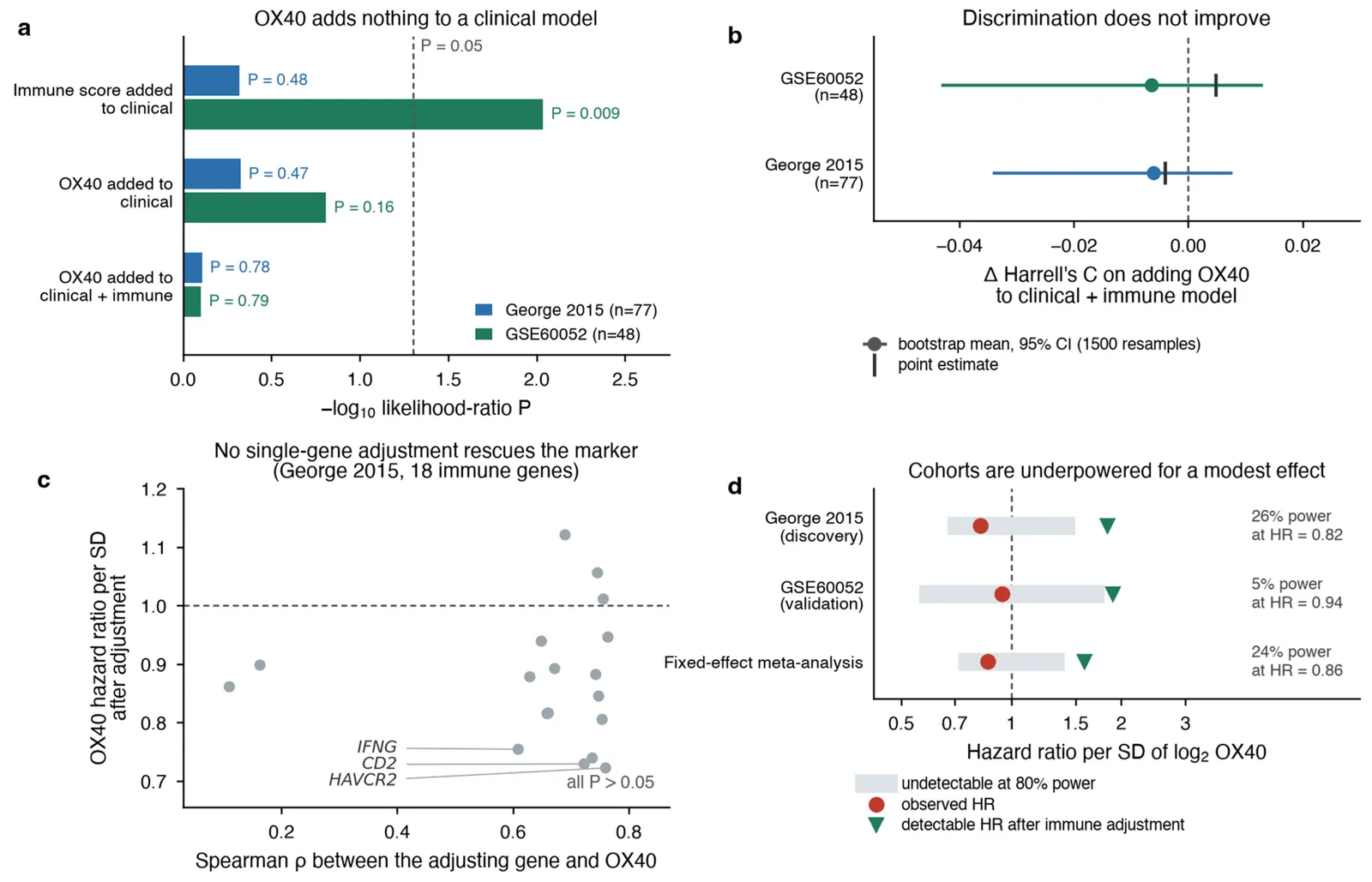
Incremental prognostic value of TNFRSF4. (**a**) Likelihood-ratio tests for each nested model addition in both cohorts; only the immune score added to the clinical model reached significance, and then only in GSE60052. (**b**) Change in Harrell’s C-index on adding TNFRSF4 to the clinical-plus-immune model, with 1500-resample bootstrap distributions and 95% percentile intervals spanning zero. (**c**) TNFRSF4 hazard ratio when each of 18 individual immune genes replaces the composite adjustment score; the effect is null throughout (minimum *p* = 0.15). (**d**) Detectable hazard ratios at 80% power for each cohort and the pooled data, before and after accounting for the cohort-specific variance inflation caused by adjusting for the immune-score covariate (factors 2.25 in George 2015 [23], 1.21 in GSE60052 and 1.91 pooled); each cohort’s observed effect is shown for reference. Colours denote cohort throughout: blue George 2015 [23], green GSE60052. In (**b**), circles with horizontal lines are bootstrap means with 95% percentile intervals and the vertical tick is the point estimate. In (**d**), grey bands span the effects undetectable at 80% power, red circles are the observed hazard ratios and green triangles the detectable hazard ratios after immune adjustment. Dashed vertical lines mark the null value.

Nested-model comparison was used to test whether OX40 contributes anything a clinician does not already know (Figure 6a). Adding TNFRSF4 to a clinical model did not improve fit in George 2015 [23] (likelihood-ratio *p* = 0.47) or GSE60052 (*p* = 0.16), and adding it to a clinical-plus-immune model was likewise uninformative (*p* = 0.78 and *p* = 0.79). In contrast, adding the immune score to the clinical model improved fit substantially in GSE60052 (*p* = 0.009), confirming that the prognostic content lies in infiltration rather than in OX40.

Discrimination told the same story (Figure 6b). Added to the clinical-plus-immune model, the change in Harrell’s C-index was −0.004 in George 2015 [23] and +0.005 in GSE60052, with 1500-resample bootstrap means of −0.006 in both cohorts and 95% intervals spanning zero (−0.034 to 0.007 and −0.043 to 0.013). Base C-indices for that model were 0.687 and 0.751, so both had reasonable baseline discrimination that OX40 did not improve. Added to the clinical model alone, without the immune score, the point estimates were larger in GSE60052 (+0.031, from 0.692 to 0.723) than in George 2015 [23] (+0.001, from 0.673 to 0.674), but neither addition improved model fit (likelihood-ratio *p* = 0.16 and *p* = 0.47) and the apparent gain in the validation cohort is of the size expected from adding any single covariate to a 48-patient, 23-event model.

Because immune markers are mutually correlated, in principle the null result could depend on which marker is used for adjustment. Repeating the analysis with each of 18 individual immune genes in place of the composite score, the TNFRSF4 hazard ratio ranged from 0.72 to 1.12 and no adjustment produced a significant OX40 effect (minimum *p* = 0.15; Figure 6c). The conclusion is therefore not an artefact of the particular infiltration score chosen.

These null results must be read against what the cohorts could detect (Figure 6d). At 80% power, George 2015 [23] could detect HR ≥ 1.50 and GSE60052 HR ≥ 1.79, with the pooled data reaching HR ≥ 1.39; the achieved power against each cohort’s own observed effect was 26%, 5% and 24%, respectively. Adjusting for a covariate correlated with TNFRSF4 inflates the variance of the TNFRSF4 coefficient, and the correlation differs between cohorts, so the inflation factor was computed separately for each. In George 2015 [23], the TNFRSF4 and immune-score covariates correlate at r = 0.745 (variance inflation factor 2.25), in GSE60052 at r = 0.419 (factor 1.21), and the pooled analysis carries the event-weighted mean of the two (factor 1.91). With these factors, the detectable effects widen to HR ≥ 1.83, HR ≥ 1.90 and HR ≥ 1.58, respectively. The event requirements likewise depend on which effect size is targeted: demonstrating the effect observed in George 2015 [23] (HR = 0.83) at 80% power would require approximately 213 events, and demonstrating the smaller pooled effect (HR = 0.86) approximately 362. The bounds are asymmetric and the observed trend is protective. At 80% power the harmful effects detectable were HR ≥ 1.50 and HR ≥ 1.79 and the protective effects HR ≤ 0.67 and HR ≤ 0.56, so the observed pooled HR of 0.86 lies well inside the undetectable range. These figures describe what the study could have detected, not what it has excluded; the study is powered only for large effects, and effects smaller than the detectable thresholds remain entirely possible. The range of effects compatible with our data is set by the reported confidence intervals, which for the pooled estimate span hazard ratios from 0.68 to 1.10 per standard deviation, and not by the power calculation. Power curves and minimum detectable effects are shown in Figure S5, and nested-model output and power calculation are given in Supplementary Tables S9 and S10.

### 2.7. Descriptive Survival Analysis in Chemotherapy Patients

Because a costimulatory marker is of clinical interest chiefly if it identifies patients who benefit from therapy, we investigated whether the survival association differed in the subset of George 2015 [23] where chemotherapy was recorded. The source clinical file records chemotherapy only as a yes/no field, without regimen, cycle number or dates, and contains no response assessment; it reports a progression-free duration with no accompanying event or censoring indicator. No treatment-anchored endpoint can therefore be constructed from it. We restricted the analysis to overall survival, the only endpoint the file records with both a time and an event indicator, and we report it as descriptive rather than as a test of treatment benefit. Among 50 patients with chemotherapy recorded (32 deaths), TNFRSF4 gave HR = 0.90 per SD (95% CI 0.62–1.30, *p* = 0.57) unadjusted, HR = 1.09 (0.76–1.56, *p* = 0.66) after adjustment for age, sex and stage, and HR = 1.46 (0.90–2.36, *p* = 0.12) after further adjustment for immune infiltration. The two adjusted models carry fewer than ten events per variable and are not interpretable as independent estimates; the unadjusted estimate is consistent with the whole-cohort result and with the null. Full model output for this subset is given in Table S11.

We deliberately do not report progression-free survival, response categories or a durable-benefit endpoint for this cohort. Each would require an event indicator, a RECIST assessment or a recorded regimen that the source data do not contain, and endpoints derived from durations alone would invite exactly the causal reading the data cannot support (Section 4.3). For the same reason, this subset is described as chemotherapy-recorded rather than platinum-exposed. Platinum-etoposide is the standard first-line regimen in SCLC, but the file does not document which agents any patient received. Whether OX40 predicts response to chemotherapy or to immunotherapy in SCLC therefore remains untested here, and testing it requires a cohort with prospectively recorded regimen, response and progression dates (Section 3).

## 3. Discussion

This multi-source analysis establishes a consistent picture of OX40 in SCLC that differs from the hypothesis with which we began. TNFRSF4 expression in bulk SCLC tissue primarily marks immune infiltration rather than tumour biology, provides no detectable incremental prognostic information beyond clinical variables and infiltration within the available sample size, and does not predict sensitivity to any agent in a 542-compound panel. The one finding that survives adjustment for infiltration, the enrichment in the POU2F3/SCLC-P subtype, is internally robust in the discovery cohort but does not replicate independently and is not separable from continuous POU2F3 expression. We therefore consider OX40 as a subtype-associated hypothesis requiring prospective evaluation, not as a biomarker ready for clinical consideration.

The evidence that bulk OX40 is a surrogate for infiltration is convergent across three independent lines. The correlation with a composite immune score reaches ρ = 0.76, which is high enough that OX40 and infiltration are close to interchangeable in a bulk model. Single-cell attribution places TNFRSF4 in T cells at roughly ten times the rate seen in malignant cells, consistently in all 20 donors; and pure tumour cell lines, which contain no immune compartment, show no drug-sensitivity signal whatsoever. Each line alone would be suggestive, but the tumour-cell-intrinsic hypothesis has to account for all three simultaneously, and it cannot. This has a direct practical consequence; any future study using bulk TNFRSF4 expression as a stratification variable would in effect be stratifying by immune infiltration, and should adjust for it explicitly or use a cell-type-resolved measurement.

The prognostic null result deserves careful rather than dismissive interpretation. Both univariable estimates and the pooled estimate of HR = 0.86 per standard deviation lay below 1 and are compatible with a real but modest benefit, whereas the adjusted estimates were directionally inconsistent. The available data do not establish a clinically actionable independent effect. At 80% power, these cohorts could detect only HR ≥ 1.50 and HR ≥ 1.79, respectively, widening to HR ≥ 1.83 and HR ≥ 1.90 once the cohort-specific variance inflation from adjusting for a correlated covariate is taken into account (factors 2.25 and 1.21). Demonstrating the effect observed in George 2015 [23] would require on the order of 213 events in that cohort, and the smaller pooled effect approximately 362 events. A nominally significant result obtained by searching for an optimal cutpoint illustrates the hazard of the alternative approach. The same data yield *p* = 0.036 when the threshold is optimised and *p* = 0.25 when that search is properly penalised. Previous reports of prognostic OX40 associations in SCLC and other tumour types should be interpreted in light of differences in cellular compartment, endpoint definition, adjustment for immune infiltration and cutpoint selection; several rely on outcome-optimised thresholds without correction for the search itself [17,18,19].

The SCLC-P association is the most biologically interesting result and also the most fragile. Its robustness within GSE60052 is not in doubt; it withstands heteroscedasticity-robust standard errors, bootstrap resampling, label permutation and partial-correlation adjustment for infiltration. Its failure to replicate in George 2015 [23]/UCologne is informative about the nature of the signal. There, a nominal elevation is present under the four-way lineage-factor scheme (*p* = 0.038) but disappears once the pan-immune score is included (*p* = 0.15). Neither continuous POU2F3 nor a POU2F3-target tuft-cell signature carries any independent association after the same adjustment, whereas the tuft signature survives adjustment comfortably in GSE60052. The replication therefore fails not for lack of a nominal signal but because the signal in the second cohort is attributable to infiltration, which is precisely the confound this study set out to test. Small subtype numbers, platform differences between RNA-seq quantifications and the known instability of transcription-factor-based subtype calls in bulk tissue remain plausible additional contributors. The collinearity between the discrete SCLC-P indicator and continuous POU2F3 expression suggests that whatever the signal is, it tracks POU2F3-driven biology on a continuum rather than a categorical subtype boundary. Mechanistically this is plausible; POU2F3-driven tumours are non-neuroendocrine and have a distinct chromatin landscape [21,22], and the dissociation from the inflamed SCLC-I state, which showed strong immune-score elevation but no TNFRSF4 elevation, indicates that OX40 in SCLC-P is not simply a consequence of these tumours being more inflamed.

Several limitations constrain the conclusions and are integral to their interpretation rather than peripheral to it. The analysis is entirely in silico and speaks to transcript abundance only: nothing in these data addresses OX40 signalling, ligand engagement, receptor function or the consequences of receptor agonism, and no result is validated at the protein level; OX40 expression measured by immunohistochemistry or flow cytometry could diverge from transcript abundance, particularly for a receptor whose surface expression is transient and activation-dependent. Both survival cohorts are small and were assembled from surgically resected tumours, which over-represent limited-stage disease relative to the extensive-stage population in which immunotherapy is used and the fully adjusted Cox models fall below ten events per variable and are reported as flagged, non-interpretable estimates. Drug-sensitivity inference rests on cell lines that lack the immune compartment in which OX40 acts, so a null result there cannot exclude an immune-mediated effect on treatment outcome in patients. The quanTIseq deconvolution is compositional and dominated by a residual compartment, so only the CD8+ T-cell concordance with marker-gene analysis is treated as supported. Single-cell detection rates are lower bounds because of transcript dropout, and the endothelial and mast-cell estimates rest on a few hundred cells. No treatment-anchored endpoint could be analysed at all. The public clinical file provides progression-free duration without an event or censoring indicator, states chemotherapy exposure only as yes/no with no regimen, and contains no response assessment, so progression-free survival, response categories and any durable-benefit endpoint would have to be imputed from durations. We judged that inadmissible and restricted the chemotherapy-recorded analysis to overall survival, reported descriptively (Section 2.7). Whether OX40 predicts response to chemotherapy or to immunotherapy in SCLC is consequently untested here rather than answered in the negative. Finally, subtype assignment from bulk expression is an approximation to a classification originally defined on protein and chromatin data. Each of these limitations has specific remedies that a follow-up study could implement. Protein-level OX40 quantification by immunohistochemistry or flow cytometry on the same tumours would test the transcript-to-protein assumption; a multi-institutional extensive-stage series of the size indicated by the power calculation, roughly 213 events, would raise every adjusted model above ten events per variable; co-culture or organoid systems containing autologous T cells would supply the immune compartment the cell-line panel lacks; prospectively recorded RECIST response, regimen and progression dates would allow the treatment-response question to be addressed with recorded endpoints; and orthogonal subtype assignment by immunohistochemistry for ASCL1, NEUROD1, POU2F3 and YAP1 would test the bulk-expression classification directly. A cohort of immunotherapy-treated patients with pretreatment tissue would additionally allow the response hypothesis stated below to be tested rather than inferred.

Beyond OX40, the analysis illustrates a general problem in bulk-tumour biomarker studies. Any immune-related gene will correlate with prognosis wherever immune infiltration itself carries prognostic weight. A survival association in bulk tissue is not evidence that the gene has a biological effect of its own; it may be an epiphenomenon of the gene acting as a proxy for the infiltrate. Distinguishing the two requires the sequence of questions applied here: which compartment expresses the transcript, whether the signal is separable from established markers of that compartment, whether the association survives several independent adjustments for it, and whether any residual effect replicates in an independently classified cohort. Answering these questions costs little beyond the data already required for the original claim, and a null residual result is informative rather than a failed experiment. It relocates the finding from tumour biology to the immune context, which changes what the marker could be used for. We suggest that reports of prognostic immune-related genes in bulk tissue should routinely include this decomposition, together with the minimum effect the cohort could have detected, so that a proxy signal is not carried forward as an independent one. The cross-tumour literature on TNFRSF4 shows exactly this pattern. High expression was associated with favourable outcome and with tumour-infiltrating immune-cell abundance in endometrial carcinoma, where the transcriptomic finding was confirmed by multiplex immunohistochemistry on tissue microarrays [13], and elevated expression was linked to immune infiltration in hepatocellular carcinoma [14]. In acute myeloid leukaemia, the direction reverses, with overexpression an independent predictor of unfavourable outcome on multivariable testing [15], and in extranodal natural killer/T-cell lymphoma, OX40 protein was detectable in 78% of cases and carried prognostic weight [16]. In none of these settings was the prognostic association tested against the infiltration association, so the inconsistency in direction may reflect differences in what the marker is proxying for rather than differences in OX40 biology.

For clinical translation, the implications are specific. OX40 agonist development in SCLC should not be guided by bulk TNFRSF4 expression, which would select for immune-infiltrated tumours rather than for a distinct biological state, and the same caution applies to using it as an enrichment criterion in trial design. The POU2F3/SCLC-P hypothesis is testable and worth testing, but it requires a cohort with adequate numbers of SCLC-P tumours, protein-level OX40 quantification, and ideally treatment-anchored endpoints in patients receiving immunotherapy. The specific hypothesis these data generate is a response hypothesis rather than a prognostic one. Because bulk TNFRSF4 tracks an activated T-cell infiltrate that also carries PDCD1 and CD8A, and because the residual subtype association points to SCLC-P, tumours combining SCLC-P assignment with a T-cell-inflamed microenvironment comprise the population in which combined OX40 agonism and PD-1/PD-L1 blockade would most plausibly be tested. Nothing in the present data predicts such a response, and this framing is offered as a hypothesis requiring pretreatment tissue, standardised response criteria and an immunotherapy-treated cohort, none of which the public datasets provide. Given that roughly 213 events would be needed to demonstrate the effect size observed in George 2015 [23], and 362 the smaller pooled effect, such a study will require multi-institutional collaboration rather than a single-centre series. The early-phase record is consistent on this point. Across four first-in-human studies of agonistic anti-OX40 antibodies in advanced solid tumours, all tolerable, single-agent activity was minimal: MEDI0562 produced two immune-related partial responses and stable disease in 44% of 55 patients [9]; MOXR0916 yielded one confirmed partial response across 34 dose-escalation and 138 expansion patients without reaching a maximum tolerated dose [10]; and BMS-986178 produced no objective response as monotherapy, with objective response rates of 0–13% across combination cohorts that the investigators judged not to exceed what nivolumab and ipilimumab alone would be expected to deliver [11]. Most informatively for biomarker development, in the ENGAGE-1 study of GSK3174998 in 138 patients, demonstrable pharmacodynamic target engagement in both peripheral blood and tumour tissue did not correlate with clinical efficacy [12]. A selection marker is therefore needed that reports on the compartment in which the receptor is engaged, which is what the present compartment attribution addresses.

## 4. Materials and Methods

### 4.1. Data Sources

Four publicly available sources were analysed (Table 1). No new data were generated and no patients were recruited. The George et al. 2015 cohort [23] was obtained from cBioPortal [25,26] (study identifier sclc_ucologne_2015) and comprises 81 tumours with expression data, of which 77 patients have known overall survival, with 48 deaths and 50 patients are recorded as having received chemotherapy. GSE60052 [27] was obtained from the Gene Expression Omnibus and comprises 79 tumours and 7 normal lung samples with RNA-seq quantification; survival data are not present in the GEO series matrix for this accession and were taken instead from the patient clinical annotation table published with the original report of the cohort (Table S1, “Chinese patient clinical summary”, in Jiang et al. 2016 [27]), which records for each sequenced patient a pathology identifier, a diagnosis date, a last-contact or death date and vital status. Samples were linked to that table through the pathology identifier embedded in the GEO sample name, the tumour sample nA corresponding to patient n. Overall survival was computed as the interval between diagnosis and last contact or death, expressed in months as days divided by 30. Forty-eight of the 79 tumours could be matched to a complete record of both dates and vital status; these 48 patients, with 23 deaths, constitute the survival cohort, and the remaining tumours were used for expression analyses only. This is the reason earlier descriptions of this dataset treated it as lacking survival information. GDSC1 and GDSC2 drug-response data were obtained from cancerrxgene.org [28,29] and matched to cell-line expression, yielding 61 SCLC lines across 542 compounds. Single-cell data were drawn from a human SCLC atlas [30] accessed through the CELLxGENE Census, selecting cells annotated as small cell lung carcinoma and retaining only non-duplicated primary data records (the is_primary_data flag, which removes cells replicated across Census datasets rather than selecting an anatomical site), giving 77,143 cells from 20 donors with both T-cell and malignant-cell annotations. The atlas samples both primary and metastatic disease: 31,540 cells (40.9%) are from lung, the remainder from lymph node (18,594), liver (9757), pleural fluid (6969), axilla (6484) and adrenal gland (3799); analyses therefore pool primary tumours with metastatic and effusion sites.

### 4.2. Processing of Expression Data

Expression values were log2-transformed with a pseudocount of one where not already on a log scale, and standardised to zero mean and unit variance within cohort before entry into regression models. This was to ensure that hazard ratios and regression coefficients are expressed per standard deviation and are comparable across cohorts. For the GDSC panel, replicate dose–response curves for the same cell line and compound were collapsed to a single median ln(IC50) before correlation, so that each cell line contributes one observation per drug and pseudo-replication is avoided. Drug × dataset combinations with fewer than 20 lines were excluded, leaving 658 tests. Immune infiltration was scored as the mean of within-cohort standardised log2 expression values, computed independently in each cohort. In George 2015 [23] the panel comprised CD2, CD247, CD3D, CD3E, CD4, CD8A, CTLA4, CXCL9, CXCL10, GZMB, IFNG, PDCD1 and PRF1; in GSE60052, where the available platform annotation differed, it comprised CD3D, CD3E, CD8A, CD74, CTLA4, CXCL9, CXCL10, GZMB, HLA-DRA, IDO1, IFNG, PDCD1 and STAT1. Both panels contain 13 genes and are dominated by cytotoxic T-cell and interferon-response transcripts, but they are not identical, so the two scores are comparable in direction and standardised scale rather than gene-for-gene. Molecular subtypes were assigned by the highest standardised score among the relevant lineage transcription factors, using ASCL1, NEUROD1, POU2F3 and YAP1 for the four-way scheme and replacing YAP1 with the immune score for the inflamed-axis scheme.

### 4.3. Survival and Regression Analysis

Overall survival was analysed by Kaplan–Meier estimation with log-rank tests and by Cox proportional hazards regression. The pre-specified primary analysis used continuous TNFRSF4 per standard deviation; median-split and data-derived cutpoint analyses are reported as secondary. For the data-derived cutpoint, all thresholds leaving at least ten patients per group were scanned for the minimum log-rank *p*-value, and the resulting *p*-value was corrected by repeating the entire search procedure on 2000 permutations of the survival labels. Four nested model specifications were fitted in each cohort: univariable; immune-adjusted (13-gene infiltration score); clinically adjusted (age, sex and stage, with chemotherapy exposure additionally included in GSE60052); and fully adjusted, containing both the clinical covariates and the immune score. Events per variable were computed for every model and models below ten were flagged as overfitted in all figures and tables. Proportional-hazards assumptions were assessed by Schoenfeld residual tests. The two univariable estimates were combined by fixed-effect inverse-variance meta-analysis on the log hazard scale.

Incremental prognostic value was assessed with likelihood-ratio tests between nested models and by the change in Harrell’s C-index, with 95% intervals obtained from 1500 bootstrap resamples. Because immune markers are mutually correlated, the adjustment was repeated with each of 18 individual immune genes in place of the composite score as a sensitivity analysis. Statistical power was computed for the Cox model given the observed number of events, and recomputed after inflating the variance by the factor implied by the correlation between TNFRSF4 and the adjustment covariate within that cohort, so that the detectable effect sizes reported reflect the adjusted rather than the marginal analysis. Because this correlation differs between cohorts, the inflation factor was derived separately for each and the pooled factor taken as the event-weighted mean; the factor for each cohort is reported with the results and in Table S10.

Two cohorts carry a treatment-status field, and they are described separately. In GSE60052, the original cohort report describes postoperative chemotherapy and notes that 13 patients had received preoperative chemotherapy [27]; the available field is a single yes/no indicator that does not distinguish treatment timing, so the covariate entering the GSE60052 clinical models is interpreted as documented chemotherapy exposure of unspecified timing, and no inference about treatment sequence is drawn from it. Separately, analyses of the George 2015 [23] chemotherapy-recorded subset were restricted to the 50 patients for whom the public clinical file documents chemotherapy as given. The public clinical file records chemotherapy only as a yes/no field, names no agent, cycle number or dates, contains no response assessment, imaging schedule or RECIST category, and reports progression-free survival as a duration with no accompanying event or censoring indicator. Constructing a progression-or-death endpoint, a response category or a durable-benefit composite from these fields would require imputing the missing event indicator from the durations themselves; we judged that inadmissible and did not fit any such model. This subset was therefore analysed for overall survival only, the single endpoint the file records with both a time and an event indicator, and it is reported descriptively (Section 2.7) rather than as a test of treatment benefit or of response to any specific regimen. Because the agents are not documented, the subset is described throughout as chemotherapy-recorded and not as platinum-exposed, even though platinum-etoposide is the standard first-line regimen for this disease.

### 4.4. Correlation, Deconvolution and Single-Cell Analysis

Associations between TNFRSF4 and immune features were quantified by Spearman correlation with Benjamini–Hochberg control of the false discovery rate applied within each analysis family. Immune cell populations were estimated by MCP-counter [31] and, as an independent approach, by quanTIseq [32]; quanTIseq fractions are compositional and were interpreted accordingly. Subtype effects were tested by Kruskal–Wallis and Mann–Whitney tests and modelled by linear regression with heteroscedasticity-robust (HC3) standard errors, with additional inference from 10,000 bootstrap resamples and 10,000 label permutations. Collinearity between the subtype indicator and continuous POU2F3 was quantified by the variance inflation factor. Partial Spearman correlations were computed on rank-residualised variables. A POU2F3-target tuft-cell signature was defined as the mean z-scored expression of ASCL2, AVIL, SOX9, TRPM5, LRMP and IGF1R, genes reported as markers of the POU2F3-driven tuft-cell-like SCLC variant [33]; the list was assembled for this study from that literature rather than taken verbatim from a validated published signature, and is therefore described as a literature-informed, study-defined signature. The seven-gene T-cell costimulatory-axis signature was constructed in the same way, as the mean z-scored log2 expression of TNFRSF4, TNFRSF9, TNFRSF18, TNFSF4, ICOS, CD27 and TNFRSF14; the list deliberately spans TNF-receptor-superfamily members, the OX40 ligand TNFSF4 and the CD28-family receptor ICOS, and is likewise a literature-informed, study-defined signature. Subtypes were assigned by the highest z-scored lineage-factor expression among ASCL1, NEUROD1, POU2F3 and YAP1 (four-way scheme) and, separately, under the inflamed-axis scheme in which the YAP1 group is replaced by an immune-defined SCLC-I state. Both assignments are reported for each cohort because the inflamed-axis scheme is derived from immune expression and therefore cannot serve as an independent test for a gene that tracks infiltration. The originally published non-negative matrix factorisation classifier was not applied: it is distributed as a fitted decomposition of its own training matrix rather than as a portable model that can be projected onto an external expression matrix, and refitting it would not carry the validation of the published classifier. The two schemes used here are therefore reported as a sensitivity analysis over assignment rules rather than as an implementation of that classifier. A POU2F3-target tuft-cell signature (ASCL2, AVIL, SOX9, TRPM5, LRMP, IGF1R) was computed as the mean z-score of its members and tested with and without adjustment for the pan-immune score. For the endothelial comparison, donor-level pairing was repeated by restriction to donors contributing at least 20 cells to both compartments.

For single-cell analysis, a cell was called TNFRSF4-positive if at least one unique molecular identifier count was observed. Positivity was summarised both pooled across cells and at the donor level, with the donor-level paired comparison between T cells and malignant cells tested by a Wilcoxon signed-rank test. Donor-level analysis was treated as primary because pooled comparisons can be driven by a single high-contributing donor. Median sequencing depth was tabulated per cell type and correlated with positivity to check whether detection differences could be explained by depth.

### 4.5. Software and Reproducibility

All analyses were performed in Python 3.13.14 (Python Software Foundation, Beaverton, OR, USA) using pandas 2.3.3, numpy 2.3.4, scipy 1.18.0, statsmodels 0.14.6 and lifelines 0.30.3 [34], with figures produced in matplotlib 3.9. quanTIseq deconvolution was run in R 4.5.3 (R Foundation for Statistical Computing, Vienna, Austria) using quantiseqr 1.18.0 (Bioconductor) [32]. Single-cell data were retrieved with cellxgene-census 1.18.0. No cell lines, reagents, devices or instruments were obtained or handled by the authors: the 61 SCLC cell lines are public entries in the Genomics of Drug Sensitivity in Cancer release files [28,29], and all drug-response and expression values were extracted computationally from those files. AI-assisted coding tools were used to support script development and editorial refinement; all analyses were executed on the original public datasets, and all code, numerical outputs, figures and interpretations were independently reviewed and verified by the authors. Random seeds were fixed for all resampling and permutation procedures. The complete supplementary tables, containing the computed result tables underlying every reported number, are provided as a spreadsheet workbook (File S3, one worksheet per table).

## 5. Conclusions

OX40/TNFRSF4 expression in bulk SCLC tissue primarily reflects the extent of tumour immune infiltration; expression was nonetheless detected in 1.03% of malignant cells, so a minor tumour-intrinsic component cannot be excluded. No independent prognostic association was detectable in either of two cohorts or in their meta-analysis; no incremental discriminatory information beyond clinical variables and infiltration was detectable within the limits of the available sample size, and it does not predict sensitivity to any of 542 compounds, including the agents used clinically in SCLC. In the subset for which chemotherapy is recorded, overall survival showed no association with TNFRSF4, but the source data document neither regimen nor response and support no inference about treatment benefit. Single-cell attribution places the transcript predominantly in T cells rather than tumour cells, consistently across all 20 donors examined.

The one association that survives adjustment for infiltration is enrichment in the POU2F3/SCLC-P subtype, which is internally robust in the discovery cohort yet fails to replicate independently and is not separable from continuous POU2F3 expression. This is the finding worth pursuing, and pursuing it requires protein-level measurement in a cohort containing adequate numbers of SCLC-P tumours with prospectively recorded treatment endpoints. The present cohorts were powered to detect only large effects, approximate hazard ratios of 1.5 or greater in the harmful direction, so smaller effects cannot be excluded. The plausible magnitude of an OX40 effect is bounded by the reported confidence intervals rather than by the power calculation, and our negative results should be considered inconclusive for modest effects rather than as evidence of absence.

## Figures and Tables

**Table 1:** Datasets analysed in this study.

Cohort	Source	Sample	Use in This Study
George et al. 2015	[23,24,25,26]	81 tumours; 77 patients with overall survival, 48 events; 50 with chemotherapy recorded	Prognosis, immune-adjusted and clinically adjusted Cox models, incremental value, descriptive survival in chemotherapy-recorded patients, subtype replication
GSE60052	[27]	79 tumours and 7 normal lungs, RNA-seq; 48 patients with survival, 23 events	Independent survival testing, immune marker correlation, deconvolution, molecular subtype assignment
GDSC1 + GDSC2	[28,29]	61 SCLC cell lines, 542 compounds, 658 drug × dataset tests	Drug sensitivity (tumour-intrinsic)
Human SCLC atlas	[30]	77,143 cells from primary and metastatic sites; 20 donors	Cellular attribution of TNFRSF4 expression

## Data Availability

All data analysed in this study are publicly available. The George et al. 2015 [23] cohort is available through cBioPortal (study identifier sclc_ucologne_2015, https://www.cbioportal.org/study/summary?id=sclc_ucologne_2015 (accessed on 27 August 2026)). GSE60052 is available from the Gene Expression Omnibus (https://www.ncbi.nlm.nih.gov/geo/query/acc.cgi?acc=GSE60052 (accessed on 27 August 2026)). GDSC1 and GDSC2 drug-response and cell-line expression data are available from the Genomics of Drug Sensitivity in Cancer resource (https://www.cancerrxgene.org (accessed on 27 August 2026)). The single-cell SCLC atlas was accessed through the CELLxGENE Census (https://cellxgene.cziscience.com (accessed on 27 August 2026)). No new data were generated.

## Supplementary Materials

The following supporting information can be downloaded at https://www.mdpi.com/article/10.3390/ijms27177823/s1.

## References

[1] Dingemans A.-M., Früh M., Ardizzoni A., Besse B., Faivre-Finn C., Hendriks L., Lantuejoul S., Peters S., Reguart N., Rudin C. (2021). Small-cell lung cancer: ESMO Clinical Practice Guidelines for diagnosis, treatment and follow-up. Ann. Oncol..

[2] Simpson K.L., Rothwell D.G., Blackhall F., Dive C. (2025). Challenges of small cell lung cancer heterogeneity and phenotypic plasticity. Nat. Rev. Cancer.

[3] Horn L., Mansfield A.S., Szczęsna A., Havel L., Krzakowski M., Hochmair M.J., Huemer F., Losonczy G., Johnson M.L., Nishio M. (2018). First-Line Atezolizumab plus Chemotherapy in Extensive-Stage Small-Cell Lung Cancer. N. Engl. J. Med..

[4] Zugazagoitia J., Osma H., Baena J., Ucero A.C., Paz-Ares L. (2024). Facts and Hopes on Cancer Immunotherapy for Small Cell Lung Cancer. Clin. Cancer Res..

[5] Qin K., Gay C.M., Byers L.A., Zhang J. (2025). The current and emerging immunotherapy paradigm in small-cell lung cancer. Nat. Cancer.

[6] Croft M., So T., Duan W., Soroosh P. (2009). The significance of OX40 and OX40L to T-cell biology and immune disease. Immunol. Rev..

[7] Willoughby J., Griffiths J., Tews I., Cragg M.S. (2017). OX40: Structure and function—What questions remain?. Mol. Immunol..

[8] Wei Y., Lu L., Zhao R., Bie Q., He B., Zhang B. (2026). The role of OX40 in cancer immunotherapy: A comprehensive review from mechanisms to clinical applications. Crit. Rev. Oncol. Hematol..

[9] Glisson B.S., Leidner R.S., Ferris R.L., Powderly J., Rizvi N.A., Keam B., Schneider R., Goel S., Ohr J.P., Burton J. (2020). Safety and Clinical Activity of MEDI0562, a Humanized OX40 Agonist Monoclonal Antibody, in Adult Patients with Advanced Solid Tumors. Clin. Cancer Res..

[10] Kim T.W., Burris H.A., de Miguel Luken M.J., Pishvaian M.J., Bang Y.J., Gordon M., Awada A., Camidge D.R., Hodi F.S., McArthur G.A. (2022). First-In-Human Phase I Study of the OX40 Agonist MOXR0916 in Patients with Advanced Solid Tumors. Clin. Cancer Res..

[11] Gutierrez M., Moreno V., Heinhuis K.M., Olszanski A.J., Spreafico A., Ong M., Chu Q., Carvajal R.D., Trigo J., Ochoa de Olza M. (2021). OX40 Agonist BMS-986178 Alone or in Combination with Nivolumab and/or Ipilimumab in Patients with Advanced Solid Tumors. Clin. Cancer Res..

[12] Postel-Vinay S., Lam V.K., Ros W., Bauer T.M., Hansen A.R., Cho D.C., Stephen Hodi F., Schellens J.H.M., Litton J.K., Aspeslagh S. (2023). First-in-human phase I study of the OX40 agonist GSK3174998 with or without pembrolizumab in patients with selected advanced solid tumors (ENGAGE-1). J. Immunother. Cancer.

[13] Ma H., Feng P.H., Yu S.N., Lu Z.H., Yu Q., Chen J. (2022). Identification and validation of TNFRSF4 as a high-profile biomarker for prognosis and immunomodulation in endometrial carcinoma. BMC Cancer.

[14] Wang D., Hu H., Ding H., Zhao H., Tian F., Chi Q. (2023). Elevated expression of TNFRSF4 impacts immune cell infiltration and gene mutation in hepatocellular carcinoma. Cancer Biomark..

[15] Gamaleldin M.A., Imbaby S.A.E. (2021). The role of tumor necrosis factor receptor superfamily member 4 (TNFRSF4) gene expression in diagnosis and prognosis of acute myeloid leukemia. Mol. Biol. Rep..

[16] Zhang H., Li Y., Zhang Q., Wang Y., Zhang S., Xing X., Shen Z., Liu H., Sang W. (2025). Prognostic value of the co-stimulatory molecule OX40 expression in Extranodal Natural Killer/T-cell Lymphoma. Ann. Med..

[17] Chen P., Wang H., Zhao L., Guo H., Zhang L., Zhang W., Sun C., Zhao S., Li W., Zhu J. (2021). Immune Checkpoints OX40 and OX40L in Small-Cell Lung Cancer: Predict Prognosis and Modulate Immune Microenvironment. Front. Oncol..

[18] Yokouchi H., Nishihara H., Harada T., Amano T., Ohkuri T., Yamazaki S., Kikuchi H., Oizumi S., Uramoto H., Tanaka F. (2021). Prognostic significance of OX40 + lymphocytes in tumor stroma of surgically resected small-cell lung cancer. OncoImmunology.

[19] Massarelli E., Lam V.K., Parra E.R., Rodriguez-Canales J., Behrens C., Diao L., Wang J., Blando J., Byers L.A., Yanamandra N. (2019). High OX-40 expression in the tumor immune infiltrate is a favorable prognostic factor of overall survival in non-small cell lung cancer. J. Immunother. Cancer.

[20] Yuan Z., Pan Y., Yuan H., Gong A. (2026). OX40/OX40L: A new target for tumor immunotherapy and its clinical research progress. Front. Oncol..

[21] Rudin C.M., Poirier J.T., Byers L.A., Dive C., Dowlati A., George J., Heymach J.V., Johnson J.E., Lehman J.M., MacPherson D. (2019). Molecular subtypes of small cell lung cancer: A synthesis of human and mouse model data. Nat. Rev. Cancer.

[22] Gay C.M., Stewart C.A., Park E.M., Diao L., Groves S.M., Heeke S., Nabet B.Y., Fujimoto J., Solis L.M., Lu W. (2021). Patterns of transcription factor programs and immune pathway activation define four major subtypes of SCLC with distinct therapeutic vulnerabilities. Cancer Cell.

[23] George J., Lim J.S., Jang S.J., Cun Y., Ozretić L., Kong G., Leenders F., Lu X., Fernández-Cuesta L., Bosco G. (2015). Comprehensive genomic profiles of small cell lung cancer. Nature.

[24] He B., Zhao R., Zhang B., Pan H., Liu J., Huang L., Wei Y., Yang D., Liang J., Wang M. (2025). Endothelial OX40 activation facilitates tumor cell escape from T cell surveillance through S1P/YAP-mediated angiogenesis. J. Clin. Investig..

[25] Cerami E., Gao J., Dogrusoz U., Gross B.E., Sumer S.O., Aksoy B.A., Jacobsen A., Byrne C.J., Heuer M.L., Larsson E. (2012). The cBio cancer genomics portal: An open platform for exploring multidimensional cancer genomics data. Cancer Discov..

[26] Gao J., Aksoy B.A., Dogrusoz U., Dresdner G., Gross B.E., Sumer S.O., Sun Y., Jacobsen A., Sinha R., Larsson E. (2013). Integrative Analysis of Complex Cancer Genomics and Clinical Profiles Using the cBioPortal. Sci. Signal..

[27] Jiang L., Huang J., Higgs B.W., Hu Z., Xiao Z., Yao X., Conley S., Zhong H., Liu Z., Brohawn P. (2016). Genomic Landscape Survey Identifies SRSF1 as a Key Oncodriver in Small Cell Lung Cancer. PLoS Genet..

[28] Yang W., Soares J., Greninger P., Edelman E.J., Lightfoot H., Forbes S., Bindal N., Beare D., Smith J.A., Thompson I.R. (2013). Genomics of Drug Sensitivity in Cancer (GDSC): A resource for therapeutic biomarker discovery in cancer cells. Nucleic Acids Res..

[29] Iorio F., Knijnenburg T.A., Vis D.J., Bignell G.R., Menden M.P., Schubert M., Aben N., Gonçalves E., Barthorpe S., Lightfoot H. (2016). A Landscape of Pharmacogenomic Interactions in Cancer. Cell.

[30] Chan J.M., Quintanal-Villalonga Á., Gao V.R., Xie Y., Allaj V., Chaudhary O., Masilionis I., Egger J., Chow A., Walle T. (2021). Signatures of plasticity, metastasis, and immunosuppression in an atlas of human small cell lung cancer. Cancer Cell.

[31] Becht E., Giraldo N.A., Lacroix L., Buttard B., Elarouci N., Petitprez F., Selves J., Laurent-Puig P., Sautes-Fridman C., Fridman W.H. (2016). Estimating the population abundance of tissue-infiltrating immune and stromal cell populations using gene expression. Genome Biol..

[32] Finotello F., Mayer C., Plattner C., Laschober G., Rieder D., Hackl H., Krogsdam A., Loncova Z., Posch W., Wilflingseder D. (2019). Molecular and pharmacological modulators of the tumor immune contexture revealed by deconvolution of RNA-seq data. Genome Med..

[33] Huang Y.-H., Klingbeil O., He X.-Y., Wu X.S., Arun G., Lu B., Somerville T.D.D., Milazzo J.P., Wilkinson J.E., Demerdash O.E. (2018). POU2F3 is a master regulator of a tuft cell-like variant of small cell lung cancer. Genes. Dev..

[34] Davidson-Pilon C. (2019). Lifelines: Survival analysis in Python. J. Open Source Softw..

