# Supplementary material for: OX40 (TNFRSF4) Tracks Immune Infiltration in Small Cell Lung Cancer and Shows a Cohort-Specific, Non-Replicated Association with the POU2F3/SCLC-P Subtype: A Multi-Source In Silico Analysis
Source: Int J Mol Sci. 2026 Aug 31;27(17):7823. doi: 10.3390/ijms27177823 (PMC13566540; doi:10.3390/ijms27177823)
Supplement: Supplementary file 1 [file ijms-27-07823-s001.zip › Supplementary_File_S2_STROBE_REMARK_Checklist.pdf]

# STROBE / REMARK Reporting Checklist

**Manuscript:** OX40 (TNFRSF4) Tracks Immune Infiltration in Small Cell Lung Cancer and Shows a Cohort-Specific, Non-Replicated Association with the POU2F3/SCLC-P Subtype: A Multi-Source In Silico Analysis

This study is a retrospective secondary in silico analysis of publicly available datasets. STROBE is used for the observational components and REMARK for the tumour-marker prognostic component. Items specific to prospective recruitment, biospecimen collection or a newly performed laboratory assay are not applicable.

## STROBE

| Item | Reporting requirement | Location | How addressed |
|---|---|---|---|
| 1 | Title and abstract | Title; Abstract | The title identifies an in silico multi-source analysis; the abstract states datasets, sample sizes, methods and principal estimates. |
| 2–3 | Background and objectives | Introduction | Biological rationale and the prognostic, predictive, immune-attribution and subtype hypotheses are stated. |
| 4 | Study design | Materials and Methods, Section 4.1 | Retrospective secondary analysis of four public data sources. |
| 5 | Setting and data sources | Section 4.1; Data Availability Statement | cBioPortal sclc_ucologne_2015, GSE60052, GDSC1/GDSC2 and CELLxGENE Census; access date reported. |
| 6 | Participants and eligibility | Sections 4.1–4.4 | Dataset-specific inclusion, survival matching, cell-line threshold and single-cell filtering are described. |
| 7–8 | Variables and measurement | Sections 4.1–4.4 | TNFRSF4, overall survival, immune scores, subtypes, drug response, cell type and clinical covariates are defined with transformations. |
| 9 | Bias | Sections 4.2–4.4; Discussion | Cutpoint-search bias, pseudo-replication, overfitting, compositional deconvolution and cohort/platform limitations are addressed. |
| 10 | Study size | Abstract; Table 1; Results | 77 survival patients in George et al.; 79 GSE60052 tumours (48 with survival); 61 cell lines; 77,143 single cells from 20 donors. |
| 11 | Quantitative variables | Sections 4.2–4.3 | Continuous per-SD exposure is primary; median split and optimized cutpoint are secondary; subtype scoring is specified. |
| 12 | Statistical methods | Sections 4.3–4.5 | Cox models, fixed-effect meta-analysis, BH-FDR, robust regression, bootstrap, permutation, partial correlation, donor pairing and power analysis are reported. |
| 13–17 | Results | Results, Sections 2.1–2.7; Figures 1–6; | Cohort flow, descriptive data, estimates with uncertainty, sensitivity analyses and limitations |

| | | | |
|---|---|---|---|
| | | Tables S1–S13 | of subgroup analyses are presented. |
| 18–21 | Discussion and generalizability | Discussion; Conclusions | Key results are interpreted cautiously; non-replication, sample size, platform and clinical-translation limits are stated. |
| 22 | Funding | Funding | No external funding. |

## REMARK

| Item | Reporting requirement | Location | How addressed |
|---|---|---|---|
| 1 | Marker, rationale and hypotheses | Introduction | OX40/TNFRSF4 rationale and hypotheses are explicit. |
| 2 | Patient characteristics and treatment | Section 4.1; Section 2.7 | Cohort sizes, events and limitations of chemotherapy fields are reported. |
| 3–4 | Assay and marker handling | Sections 4.1–4.2 | Public expression platforms, log transformation, within-cohort standardization and continuous/secondary cutpoint handling are described. |
| 5 | Study design and endpoint | Sections 4.1 and 4.3 | Overall survival is the prognostic endpoint; the analysis is retrospective and secondary. |
| 6–8 | Statistical analysis | Sections 4.3–4.5 | Univariable and adjusted Cox models, EPV checks, proportional-hazards checks, meta-analysis, nested models and resampling are specified. |
| 9 | Effect estimates | Section 2.1; Figure 1; Table S1 | Hazard ratios, 95% confidence intervals and p values are reported. |
| 10 | Validation and robustness | Sections 2.1, 2.5–2.6; Figures 1, 5–6 | Independent cohort estimates, non-replication, residual adjustment, bootstrap/permutation and incremental-value analyses are reported. |
| 11 | Limitations and interpretation | Discussion; Conclusions | The null prognostic result is bounded by confidence intervals and limited power; subtype findings are presented as hypothesis-generating. |

Ethics note: no new participants or specimens were recruited or collected. The study used public, de-identified data; the original studies were responsible for participant consent and ethical approvals.
