# Supplementary material for: OX40 (TNFRSF4) Tracks Immune Infiltration in Small Cell Lung Cancer and Shows a Cohort-Specific, Non-Replicated Association with the POU2F3/SCLC-P Subtype: A Multi-Source In Silico Analysis
Source: Int J Mol Sci. 2026 Aug 31;27(17):7823. doi: 10.3390/ijms27177823 (PMC13566540; doi:10.3390/ijms27177823)
Supplement: Supplementary file 1 [file ijms-27-07823-s001.zip › Supplementary_Materials.pdf]

Supplementary Materials

OX40 (TNFRSF4) Tracks Immune Infiltration in Small Cell Lung Cancer and Shows Non-Replicating Enrichment in the POU2F3/SCLC-P Subtype: A Multi-Source In Silico Analysis

Supplementary Figures

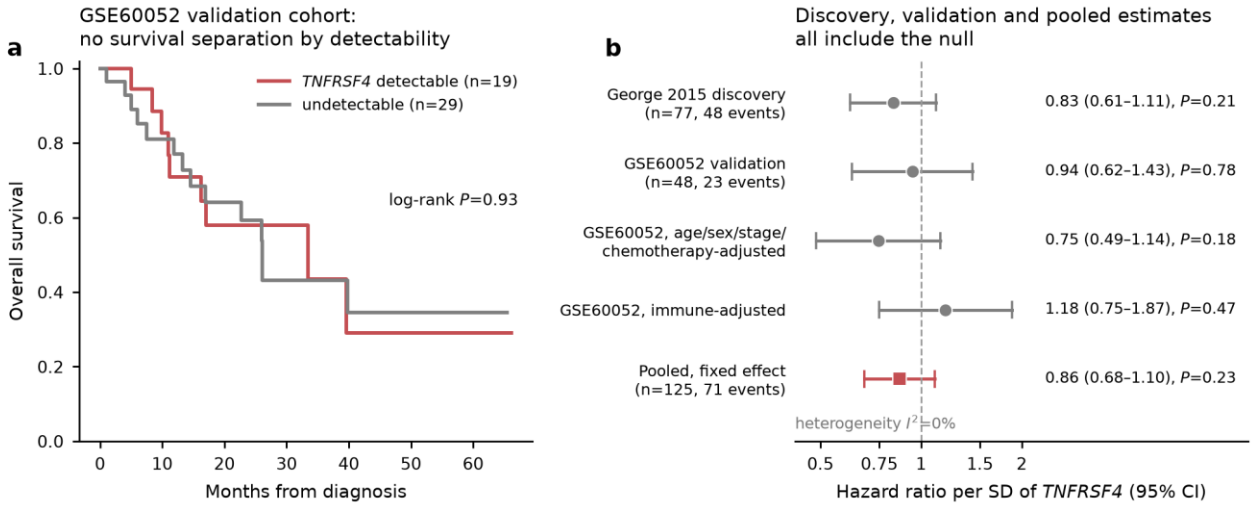

Figure S1. External validation in GSE60052, Kaplan-Meier curves and pooled hazard-ratio forest plot

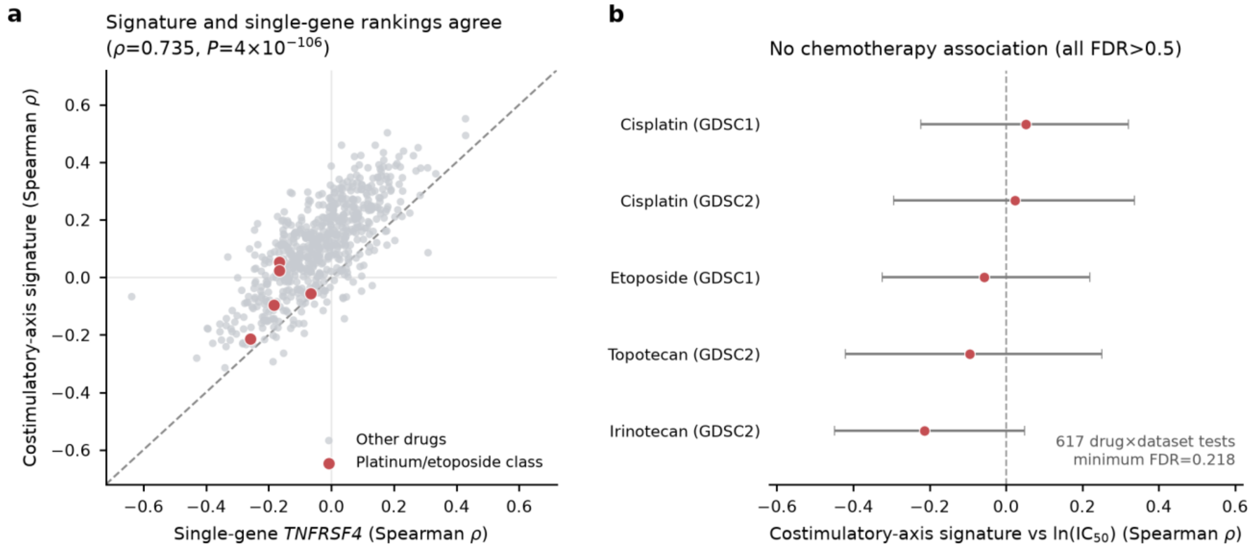

Figure S2. Seven-gene T-cell costimulatory-axis signature versus GDSC drug sensitivity

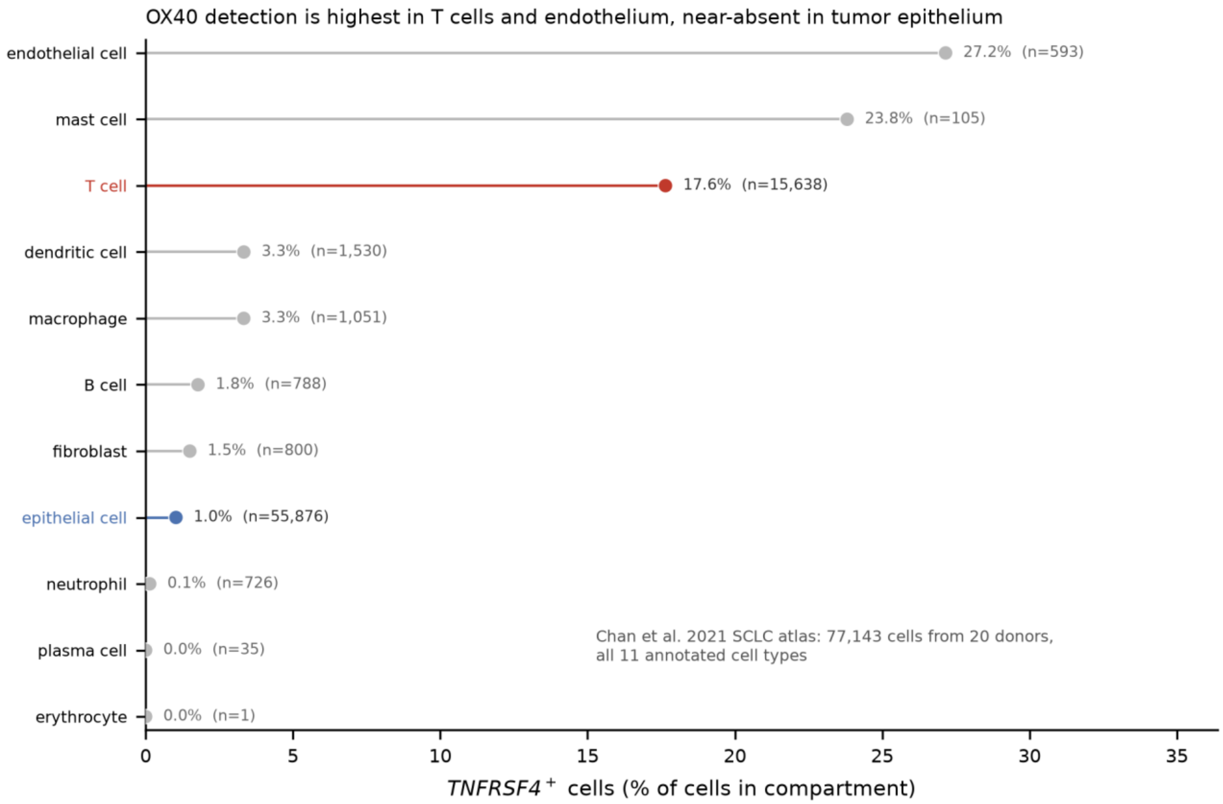

Figure S3. TNFRSF4 positivity across all 11 annotated single-cell populations

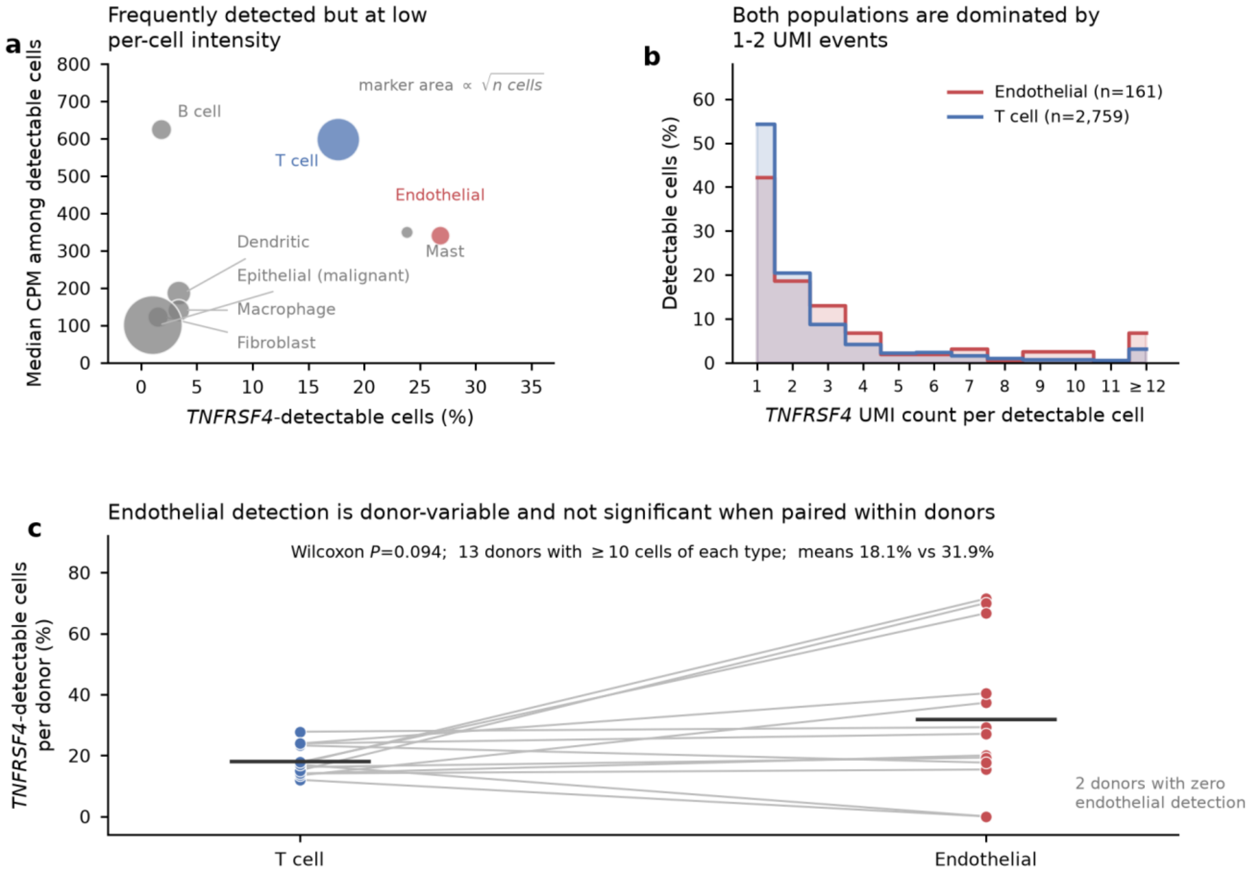

Figure S4. Per-cell intensity and donor-level reproducibility of endothelial detection

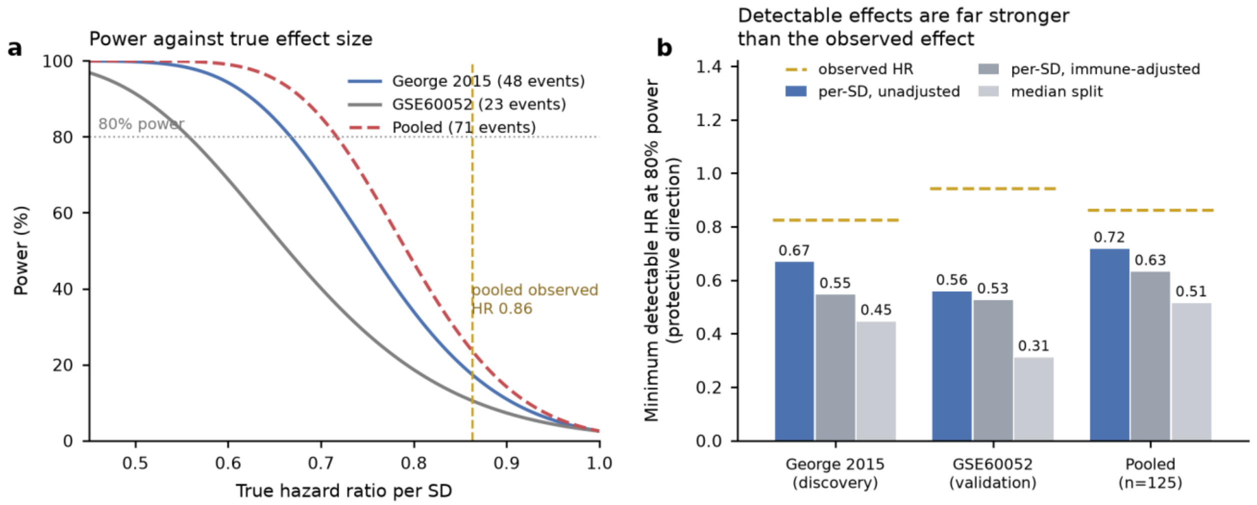

Figure S5. Power curves and minimum detectable effect sizes

Supplementary Tables

All supplementary tables are provided as sheets of Supplementary_Tables_S1-S13.xlsx (sheet names S1_Cox_models, S2_Drug_TNFRSF4, …, S13_Endothelial_matched) and additionally as individual comma-separated files Table_S1.csv – Table_S13.csv.

Table S1. Full Cox proportional-hazards output for both cohorts and all model specifications, with events-per-variable diagnostics.

Table S2. Single-gene TNFRSF4 versus ln(IC50) across 658 drug×dataset combinations in GDSC1 and GDSC2, one observation per cell line; uncollapsed estimates retained for comparison.

Table S3. Seven-gene TNF-receptor costimulatory signature versus ln(IC50), 618 drug×dataset combinations.

Table S4. Spearman correlations of TNFRSF4 with immune marker genes and MCP-counter scores in GSE60052, with FDR.

Table S5. quanTIseq cell-type fractions: correlations with TNFRSF4 and mean fractions across samples.

Table S6. Per-cell-type TNFRSF4 detection rates, per-cell intensity measures and median sequencing depth in the single-cell atlas.

Table S7. Depth-adjusted logistic model of TNFRSF4 detection by cell type, T cells as reference category.

Table S8. Subtype analyses: regression models for the SCLC-P indicator and continuous POU2F3, partial correlations, and replication and permutation tests in the independent cohort.

Table S9. Nested-model likelihood-ratio tests, C-index changes with bootstrap confidence intervals, and per-immune-marker sensitivity analysis.

Table S10. Power analysis: achieved power, minimum detectable hazard ratios under three specifications, and variance-inflation adjustment.

Table S11. Descriptive overall-survival models in the subset of George 2015 with chemotherapy recorded, with events-per-variable diagnostics.

Table S12. Independent subtype replication in George 2015 under the four-way ASCL1/NEUROD1/POU2F3/YAP1 scheme, with immune-adjusted subtype, lineage-factor and tuft-signature models in both cohorts.

Table S13. Donor-matched endothelial versus T-cell TNFRSF4 comparison restricted to donors contributing at least 20 cells per compartment.

File S1. Reproducibility archive (Supplementary_File_S1_reproducibility_archive.zip) containing all analysis code, intermediate result tables and figure generators.

File S2. STROBE and REMARK reporting checklists (Supplementary_File_S2_STROBE_REMARK_checklist.md).
